# Orchid genome evolution and trait innovation

**DOI:** 10.1111/jipb.70315

**Published:** 2026-06-25

**Authors:** Meng‐Yao Zeng, Cheng‐Yuan Zhou, Linying Wang, Jie Gao, Weilun Yin, Dong‐Hui Peng, Siren Lan, Feng‐Xi Yang, Zhong‐Jian Liu

**Affiliations:** ^1^ Key Laboratory of National Forestry and Grassland Administration for Orchid Conservation and Utilization Fujian Agriculture and Forestry University Fuzhou 350002 China; ^2^ College of Forestry Fujian Agriculture and Forestry University Fuzhou 350002 China; ^3^ Guangdong Key Laboratory of Ornamental Plant Germplasm Innovation and Utilization, Institute of Environmental Horticulture Guangdong Academy of Agricultural Sciences Guangzhou 510640 China; ^4^ College of Biological Sciences and Technology Beijing Forestry University Beijing 100083 China; ^5^ Guangzhou Institute of Forestry and Landscape Architecture Engineering Technology Research Center of National Forestry and Grassland Administration for Orchid Conservation and Utilization Guangzhou 510405 China

**Keywords:** adaptive radiation, diversification, genome evolution, multi‐omics, Orchidaceae, phylogenomics

## Abstract

Orchidaceae, one of the largest and most morphologically diverse angiosperm families, showcases unique evolutionary adaptations in morphology, ecology, and function. Recent advances in molecular and genomic research have greatly reshaped our understanding of orchid evolution, revealing how genome dynamics, ecological interactions, and developmental plasticity jointly shaped their exceptional diversification. Phylogenomic frameworks derived from various genomic datasets have reconstructed the evolutionary history, revealing the influence of geological, climatic, and biotic factors on ancient divergences and global distributions. Comprehensive genomic studies have uncovered substantial variation in genome size, structure, and composition, largely driven by repetitive elements and whole‐genome duplication events that facilitated adaptive radiations. Key innovations, including epiphytism, mycoheterotrophy, and deceptive pollination, are linked to gene family evolution and modifications in pathways related to CAM photosynthesis, mycorrhizal symbiosis, and floral morphogenesis. Integrative multi‐omics approaches further illuminate mechanisms underlying speciation hotspots, coevolution with pollinators and fungi, and the molecular basis of developmental diversity. Overall, this review synthesizes current genomic, phylogenetic, and functional insights into orchid evolution, providing a theoretical foundation and future research framework for understanding their molecular diversification.

## Graphical abstract

Orchids became one of the world's most diverse plant groups through genome‐driven innovations, unique relationships with fungi and pollinators, and remarkable adaptability. This review explains the origins of orchids and the evolution of their distinctive life forms, flowers, and ecological strategies and highlights promising directions for future research.

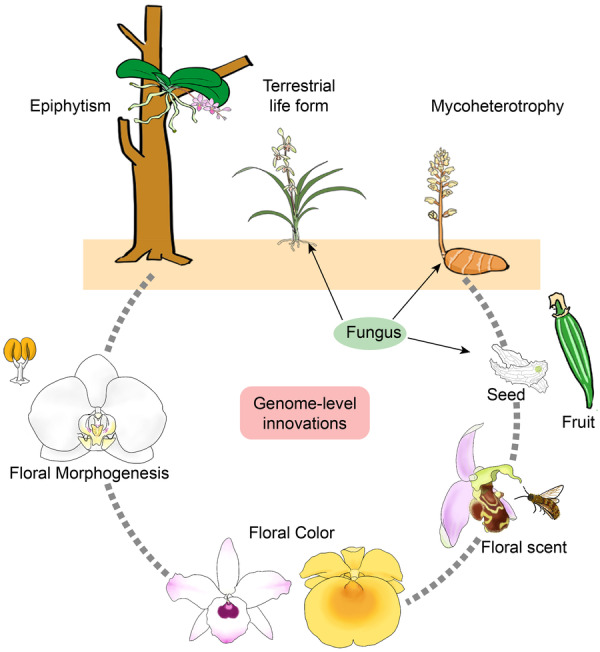

## INTRODUCTION

Taxonomic groups with exceptional species richness, pronounced phenotypic disparity, and deep evolutionary histories are widely recognized as excellent models for studying the drivers of speciation and evolutionary innovation (Lamichhaney et al., 2015; Eiserhardt et al., 2017; Tietje et al., 2022). One such lineage is Orchidaceae, which is one of the largest and most diverse families in flowering plants, comprising approximately 700 genera and 31,000 species, nearly 10% of all flowering plants (Govaerts, 2024). As a quintessential “evolutionary marvel”, orchids exhibit extraordinary morphological specialization and functional adaptations. Molecular and fossil evidence suggest that the orchid family originated in the Late Cretaceous, approximately 76–112 million years ago (Ma) (Ramírez et al., 2007; Gustafsson et al., 2010; Givnish et al., 2016; Pérez‐Escobar et al., 2024). Subsequently, orchids evolved diverse life forms, including epiphytic, terrestrial, and mycoheterotrophic lifestyles (holomycotrophy and mixotrophy) (Gravendeel et al., 2004; Selosse et al., 2022), accompanied by the evolution of complex mycorrhizal symbioses (Li et al., 2022a; Perotto and Balestrini, 2024), remarkable morphological diversity, and unique deceptive pollination strategies (Gaskett, 2011; Scaccabarozzi et al., 2018; Ackerman et al., 2023). The interplay between these biological innovations and historical plate tectonics has driven the extensive adaptive radiation of orchids (Givnish et al., 2015, 2016; Baguette et al., 2020).

Orchidaceae exhibits a near‐global distribution, occupying nearly all terrestrial ecosystems except polar ice sheets and hyperarid deserts, with biodiversity hotspots concentrated in tropical and subtropical humid zones (Pridgeon et al., 1999–2014; Vitt et al., 2023). The most species‐rich areas worldwide occur in the Andes, eastern Himalayas, Southeast Asian archipelago, and Mesoamerican cloud forests (Givnish et al., 2016; Pérez‐Escobar et al., 2017a, 2024; Parra‐Sanchez et al., 2023; Vitt et al., 2023). In temperate regions, terrestrial groups (e.g., *Cypripedium*, *Orchis*, and *Dactylorhiza*) predominate and exhibit distinctive adaptations to seasonal climatic regimes through phenological synchrony and the development of an underground storage organ (Tsiftsis et al., 2019; Liao et al., 2024).

Orchidaceae is characterized by several diagnostic synapomorphies, including zygomorphic flowers (except Apostasioideae), typically resupinate by a 180° twist to position the specialized median petal (lip) inferiorly; androecium and gynoecium fused into a gynostemium (column), and pollen grains aggregated into pollinia; and minute, dust‐like seeds lacking endosperm with undifferentiated embryos that require mycorrhizal fungi for germination (Pridgeon et al., 1999–2014; Arditti and Ghani, 2000). Variations in the basic floral structure of orchids underpin the extraordinary diversity in floral color, shape, and size (ranging from less than 5 mm in *Platystele* to more than 35 cm in *Bulbophyllum echinolabium*) (Wiśniewska et al., 2019; Patrón, 2022). The lip has evolved diverse morphologies functioning as pollinator traps, including sexual deception through mimicry of female insects (e.g., *Ophrys* and *Myrmechila*) (Pouyanne, 1917; Bower, 2007), brood‐site mimicry via pouch‐like structures (e.g., *Paphiopedilum* and *Cypripedium*) (Shi et al., 2007, 2009; Li and Luo, 2009), generalized food deception employing false nectar signals (e.g., *Cypripedium* and *Cymbidium*) (Bänziger et al., 2005; Yu et al., 2008; Li et al., 2012), and Batesian floral mimicry (e.g., *Traunsteinera* mimicking *Knautia* and *Diuris* mimicking *Daviesia*) (Jersáková et al., 2016; Scaccabarozzi et al., 2018). Some orchid species emit scent compounds that mimic food rewards, such as floral or fruity scents and decaying‐matter odors (Phillips et al., 2009; Nakahira et al., 2018), as well as female insect sex pheromones (Hetherington‐Rauth and Ramírez, 2016; Dormont et al., 2019; Perkins et al., 2023). Together, these features highlight the central role of pollinator‐mediated selection in shaping floral innovation in orchids.

Orchids are a vital component of the floriculture industry due to its unique floral morphology, vibrant coloration, and elegant fragrance. Over the past two decades, key commercial genera included *Phalaenopsis*, *Dendrobium*, *Oncidium*, and *Cymbidium*, which dominated global markets through selective breeding and horticultural innovation (Hegde, 2020; Zhang et al., 2022; Yang et al., 2024). Orchids represent an important source of medicinal resources and have been historically utilized in traditional medicinal systems such as traditional Chinese medicine, Ayurvedic medicine, and South American ethnomedicine. In China, approximately 42 genera of orchids are used in traditional medicine. Modern pharmacological investigations have demonstrated that bioactive compounds derived from medicinal orchids exhibit potent immunomodulatory, anti‐inflammatory, antitumor, and antioxidant activities (Teoh, 2019; Guo et al., 2024). Consequently, the orchid industry demonstrates substantial economic value in both horticultural and pharmaceutical sectors.

Recent advancements in research technologies and analytical methodologies have significantly propelled progress in orchid research. Over the past decade, integrative approaches combining phylogenetics, biogeography, population genetics, and multi‐omics analyses (including genomics, transcriptomics, and metabolomics) have shed new light on the origin and evolution of orchids. Meanwhile, studies have increasingly focused on adaptive radiation, molecular mechanisms of morphogenesis, coevolution between flowers and pollinators, and orchid‐fungi symbiosis patterns. These investigations have provided novel insights and conceptual frameworks for understanding plant evolution and broader biological processes in nature. In this review, we synthesize current advances in orchid evolutionary research, spanning macroevolutionary patterns, genome dynamics, and key innovations in life form and floral diversity. We highlight how integrative approaches, including phylogenomics, biogeography, multi‐omics, and functional studies, have deepened our understanding of orchid diversification, ecological partnerships, and trait evolution. By bringing together these perspectives, we aim to provide a comprehensive framework that situates recent progress, identifies remaining challenges, and points to future opportunities in orchid evolutionary biology and applied research.

## DIVERSITY AND PHYLOGENETICS OF ORCHIDACEAE

### Traditional molecular markers

**Figure 1:**
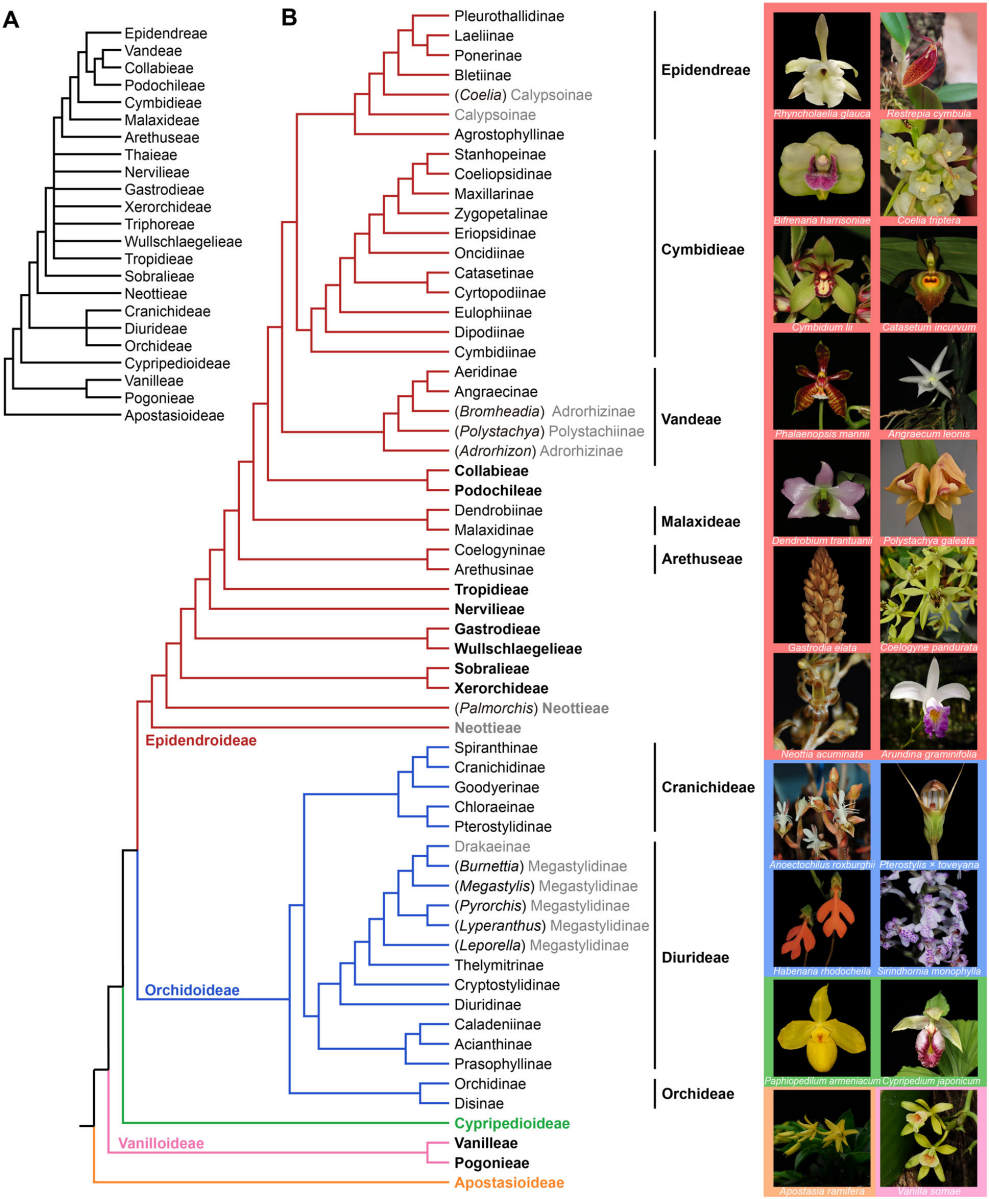
Phylogenetic relationships in Orchidaceae **(A)** Phylogeny of Orchidaceae tribes, adapted from Chase et al. (2015). **(B)** Schematic phylogeny of Orchidaceae based on the latest phylogenomic framework (Pérez‐Escobar et al., 2024). Sampling includes 17 of 22 tribes and 40 of 49 subtribes and therefore does not represent all lineages. Colored labels represent the five subfamilies of Orchidaceae; bold text denotes tribes, non‐bold text denotes subtribes; names in parentheses denote genera, and gray labels denote non‐monophyletic clades.

Phylogenetics provides the fundamental framework for understanding the origin and diversification of lineages. The molecular systematics of Orchidaceae originated in the 1990s, pioneered by the use of Sanger sequencing of traditional molecular markers, including nuclear ITS and *Xdh*, plastid *matK*, *psaB*, *rbcL*, *trnL‐F*, and *ycf1*. The phylogenetic framework of Orchidaceae was consistently resolved into five subfamilies and supported the relationship of (Apostasioideae (Vanilloideae (Cypripedioideae (Orchidoideae, Epidendroideae)))) (Chase et al., 1994, 2003; Cameron et al., 1999; Freudenstein et al., 2004; Chase, 2005). Based on early research, Chase et al. (2015) provided a synthetic revision of the orchid phylogenetic system (Figure 1A). Key modifications within Epidendroideae include the recognition of three new tribes: Thaieae, Xerorchideae, and Wullschlaegelieae. The placement of Gastrodieae within the neottioid grade was confirmed, with Neottieae identified as the basal clade of this subfamily. Several subtribes, including Vanillinae, Pogoniinae, and Collabiinae, were elevated to tribal rank, whereas others were reassigned. Phylogenetic relationships among most tribes and subtribes were also resolved. This updated system, comprising 22 tribes, 49 subtribes, and 736 genera, serves as a foundational framework for contemporary phylogenetic research in Orchidaceae. The phylogeny of Chinese orchids reconstructed by Li et al. (2016) was largely congruent with the system proposed by Chase et al. (2015), leading to a revised phylogenetic framework for Chinese Orchidaceae comprising five subfamilies, 17 tribes, and 21 subtribes.

Other studies primarily focused on resolving phylogenetic relationships at lower taxonomic levels, particularly tribes, subtribes, and genera (Tsai et al., 2010; Neubig et al., 2012; Xiang et al., 2013; Pérez‐Escobar et al., 2017b; Li et al., 2019a; Hu et al., 2020; Zhang et al., 2021a). These studies employed diverse combinations of molecular markers tailored to specific taxonomic groups, thereby resolving numerous longstanding controversies in morphology‐based classification. However, traditional molecular markers often provide limited informative sites, which constrains their ability to infer robust phylogenetic relationships in lineages characterized by rapid radiation, reticulate evolution, ancient hybridization events, or adaptive trait diversification.

### Plastome dataset

The plastome represents an ideal molecular marker due to its distinctive genetic features, such as uniparental inheritance, high gene content, relatively conserved structure, and moderate evolutionary rates (Davis et al., 2014; Niu et al., 2017). Givnish et al. (2015) were the first to employ plastome data for phylogenetic reconstruction in Orchidaceae, sampling only 39 species yet achieving higher nodal support than trees based on traditional molecular markers. Subsequent studies expanded taxon sampling to resolve relationships among tribes and subtribes (Table 1; Li et al., 2019b; Kim et al., 2020; Serna‐Sánchez et al., 2021). Additionally, plastid phylogenomic studies have helped clarify controversial intergeneric and interspecific relationships, particularly within genera or species complexes with morphologically ambiguous boundaries caused by rapid radiation or convergent evolution (Barrett et al., 2018; Lallemand et al., 2019; Liu et al., 2020; Chen et al., 2020a, 2024a; Tu et al., 2021; Guo et al., 2021a, 2025; Wu et al., 2023, 2024). In recent years, the plastome data have become a cornerstone in evolutionary studies of Orchidaceae due to their high phylogenetic resolution. These data have facilitated taxonomic revisions of complex lineages and offered a robust framework for investigating macroevolution, biogeography, and adaptive evolution.

**Table 1:** Phylogenetic relationships of Orchidaceae were constructed based on different molecular markers

Year	Molecular marker	No. of samples	Reference
2015	Protein‐coding genes of plastomes	39	Givnish et al., 2015
2015	315 single‐copy genes by transcriptome	10	Deng et al., 2015
2019	Protein‐coding genes of plastomes and the mitochondrial genome	74	Li et al., 2019b
2020	Protein‐coding genes of plastomes	124	Kim et al., 2020
2021	Protein‐coding genes of plastomes	264	Serna‐Sánchez et al., 2021
2021	Orchidaceae963 bait set	28	Eserman et al., 2021
2021	294 low‐copy nuclear genes using the Angiosperms353	264	Pérez‐Escobar et al., 2021
2022	Transcriptome	69	Wong and Peakall, 2022
2023	1,450 low‐copy nuclear genes	610	Zhang et al., 2023a
2024	Angiosperms353 loci, ITS, and *matK*	1,940	Pérez‐Escobar et al., 2024
2024	Orchidinae‐205	79	Veltman et al., 2024
2025	Angiosperms353	148	Anthoons et al., 2025

The plastomes of Orchidaceae exhibit substantial size variation, ranging from 19,047 bp (*Epipogium roseum*) to 212,668 bp (*Cypripedium subtropicum*) (Schelkunov et al., 2015; Guo et al., 2021b). In heterotrophic groups (e.g., *Gastrodia*, *Corallorhiza*, and *Lecanorchis*), extensive gene loss has occurred, particularly among photosynthesis‐related genes, resulting in plastome structures and evolutionary rates that differ markedly from those of the autotrophic species. Consequently, these lineages are prone to long‐branch attraction in phylogenetic analyses (Lam et al., 2018; Wen et al., 2022). Furthermore, challenges such as incomplete lineage sorting and hybridization complicate phylogenetic inference (Bogarín et al., 2018; Wu et al., 2023; Ji et al., 2024a), necessitating the integration of nuclear and transcriptomic data to fully resolve the evolutionary history of Orchidaceae.

### Mitochondrial genomes

Plant mitochondrial genomes are typically large, characterized by lower synonymous substitution rates than their nuclear and plastid counterparts, and enriched in expanded non‐coding regions, features that render them informative for phylogenetic inference (Li et al., 2019b). Their high content of repetitive sequences leads to frequent structural rearrangements, which in turn necessitate the integration of second‐ and third‐generation sequencing approaches to achieve accurate assemblies (Zou et al., 2022). In Orchidaceae, mitochondrial genomics remains in its early stages, with most efforts focusing on structural characterization of individual species (Chen et al., 2020b; Ke et al., 2023; Yang et al., 2023a; Zheng et al., 2024; Zhao et al., 2024a; Liu et al., 2025). Nevertheless, mitochondrial data have begun to be used in phylogenetic reconstructions. Among existing studies, Li et al. (2019b) conducted the most representative analysis to date, employing 38 mitochondrial coding sequences (mtCDS) from 74 orchid species. Their results strongly supported the division of Orchidaceae into five subfamilies, although the phylogenetic positions of Vanilloideae and Cypripedioideae were inverted relative to plastid‐based phylogenies. Importantly, the placements of several mycoheterotrophic lineages, including Epipogiinae, Gastrodieae, Nerviliinae, and Tropidieae, were well resolved. Other investigations have been more limited in scope, focusing on specific groups (Wang et al., 2023a, 2023b, 2025a; Qin et al., 2025). Wang et al. (2025a) similarly highlighted the utility of mitochondrial data in resolving relationships among mycoheterotrophic orchids. Although topological conflicts have been observed between mitochondrial‐ and plastid‐derived phylogenies, both exhibit strong statistical support, likely reflecting differences in evolutionary rates across genomes. Collectively, mtCDS data provide an important complement to traditional plastid datasets, offering critical insights into complex evolutionary histories, particularly within mycoheterotrophic lineages where plastid data alone are insufficient.

### Single/low copy nuclear genes

Recent advances in high‐throughput sequencing have significantly propelled phylogenetic research in Orchidaceae. Recent studies have focused on large‐scale datasets, employing methods such as transcriptome sequencing and target capture to acquire hundreds to thousands of single/low‐copy nuclear genes. These approaches have greatly improved phylogenetic resolution and helped overcome limitations associated with plastid‐based inference, including uniparental inheritance and the difficulty of resolving histories shaped by incomplete lineage sorting, thereby establishing a robust evolutionary framework spanning subfamilial, tribal, and population levels.

Transcriptome‐derived single/low‐copy nuclear genes have greatly improved phylogenetic resolution in Orchidaceae. Early transcriptomic analyses using hundreds of orthologous or single/low‐ copy genes consistently recovered the major subfamily relationships and clarified the placement of heterotrophic lineages (Deng et al., 2015; Unruh et al., 2018; Wong and Peakall, 2022). More recently, large‐scale datasets, such as the 1,450 low‐copy nuclear genes sampled from 610 species by Zhang et al. (2023a), have further resolved relationships across tribes and subtribes. These studies demonstrate that transcriptome‐based nuclear genes provide a robust and reliable framework for reconstructing orchid phylogeny.

Target capture is a high‐throughput sequencing technique that uses custom RNA probes to enrich and sequence hundreds to thousands of specific single/low‐copy nuclear gene loci through complementary hybridization to target sequences. Three probe sets have been developed or applied in Orchidaceae (Table 1): (1) Angiosperms353 probe targets 353 universal angiosperm nuclear genes, suitable for broad taxonomic classification (Johnson et al., 2019); (2) Orchidaceae963 probe targets 963 low‐copy nuclear genes in Orchidaceae, optimized for family‐level resolution (Eserman et al., 2021); and (3) Orchidinae‐205 probe targets 205 markers in the Orchidinae subtribe (174 low‐copy nuclear genes + 31 candidate genes related to glucomannan biosynthesis), offering subtribe‐specific analysis (Veltman et al., 2024). These datasets have improved locus recovery, resolved relationships from subfamily to subtribal and generic levels, and have been widely used to construct large‐scale orchid phylogenies (Pérez‐Escobar et al., 2021, 2024; Anthoons et al., 2025). Pérez‐Escobar et al. (2024) integrated Angiosperms353 data with ITS and *matK* sequences to generate the largest orchid tree of life to date, providing a comprehensive framework for studying orchid origins and diversification (Figure 1B). Although target‐capture studies in orchids remain relatively limited compared with plastid and transcriptomic datasets, they hold great potential for resolving complex evolutionary histories shaped by rapid radiation, hybridization, and incomplete lineage sorting.

## THE BIOGEOGRAPHIC ORIGIN AND DISPERSAL ROUTES OF ORCHIDACEAE

### The origin of Orchidaceae

The phylogenetic framework of Orchidaceae has become increasingly stable and well supported, bolstered by fossil evidence and molecular clock analyses, thereby providing a robust foundation for understanding its origin and evolutionary history. Integrating multiple factors, including geological events, global climatic shifts, temperature fluctuations, sea‐level changes, and coevolution with associated biota, enables a more systematic biogeographic investigation of the geographic origin, dispersal routes, and global diversification of Orchidaceae.

The fossil record of Orchidaceae is exceedingly scarce but remains essential for calibrating molecular clocks and inferring divergence times. Notable examples include *Meliorchis caribea* from Dominican amber, which preserved a pollinarium of Goodyerinae attached to an extinct stingless bee and dated to ca. 15–20 Ma (Ramírez et al., 2007). Leaf fossils of *Dendrobium* and *Earina* from the Early Miocene (ca. 20 Ma) of New Zealand represented rare examples of preserved orchid leaves and provided evidence for the early distribution of these lineages at the subfamilial level (Conran et al., 2009). *Succinanthera baltica*, an orchid anther cap with pollinia and caudicular viscin of Epidendroideae, preserved in Baltic amber and dated to about 40–45 Ma, was one of the oldest orchid fossils known (Poinar and Rasmussen, 2017). Numerous subfossil seeds of *Epipactis* and *Dactylorhiza* were found in early Holocene sediments in northern Poland, representing the first fossil record of orchid seeds (Gołaszewska et al., 2019). In addition, sporadic pollen fossils have been tentatively assigned to Orchidaceae, though their taxonomic placements remain contentious due to limited preservation and diagnostic characteristics (Freudenstein, 2025). Although limited, these fossils provide critical calibration points for molecular dating and have substantially refined temporal estimates of orchid diversification.

More importantly, orchid fossils are highly informative phylogenetically and have played a central role in reshaping hypotheses of both the age and ancestral area of the family. Before reliable fossil evidence became available, the hypothesis that Asia was the ancestral region of Orchidaceae was mainly supported by the distribution of Apostasioideae, the basal lineage of the family, which occurs largely in equatorial Asia (Garay, 1972). Subsequently, the discovery of orchid fossils has significantly advanced the field of orchid historical biogeography. Based on the fossil *Meliorchis caribea*, Ramírez et al. (2007) dated the origin of crown Orchidaceae to the Late Cretaceous (approximately 76–84 Ma) using relaxed molecular clock calibration. Gustafsson et al. (2010) similarly inferred that the most recent common ancestor of extant orchids arose around 77 Ma (95% confidence interval: 63–92 Ma) and that the divergence between Orchidoideae and Epidendroideae occurred around 61 Ma, coinciding with the global cooling that followed the Early Eocene Climatic Optimum. Givnish et al. (2015, 2016, 2018) conducted extensive studies on the origin and diversification of Orchidaceae. Using traditional molecular markers and up to 17 fossil calibrations, they inferred that Orchidaceae originated in Australia around 112 Ma, when Australia, Neotropics, and Antarctica were still connected as part of Gondwana. The crown group of Orchidaceae was estimated to have diversified around 90 Ma, with early dispersal facilitated by the paleo‐Antarctic land bridge, leading to its contemporary disjunct distribution spanning Australia and the Neotropics. The estimated divergence times of the basal lineages are consistent with the chronology of Gondwanan breakup. The ancestors of Vanilloideae, Cypripedioideae, Orchidoideae, and Epidendroideae were inferred to have originated in the Neotropics between 84 and 64 Ma (Givnish et al., 2016). Subsequent studies utilizing diverse datasets have increasingly converged in their estimates, suggesting that the crown age of Orchidaceae likely falls within the range of 80–90 Ma (Li et al., 2019b; Kim et al., 2020; Serna‐Sánchez et al., 2021).

Recent genome‐scale analyses have further refined these estimates and challenged earlier biogeographic hypotheses. Based on a dataset of 1,450 single‐copy genes and incorporating 14 fossil calibrations (including three orchid fossils) and two secondary calibration points, Zhang et al. (2023a) estimated an older crown age of approximately 101.5 Ma (97.08–102.56 Ma). Their analysis also estimated the divergence time of the Orchidoideae–Epidendroideae clade at around 77.7 Ma (74.5–79.7 Ma), with most subtribes diversifying between 52 and 29 Ma. In contrast, Pérez‐Escobar et al. (2024) inferred a Late Cretaceous origin (83 ± 10 Ma) but proposed a Laurasian rather than Australian origin for the family. According to their reconstruction, orchids likely achieved global distribution through long‐distance dispersal, while the extant distributions of basal subfamilies such as Apostasioideae and Cypripedioideae represent relictual patterns following the extinction of higher‐latitude lineages during Oligocene global cooling. Overall, fossil‐calibrated phylogenomic studies now converge on a Late Cretaceous origin for Orchidaceae but differ in reconstructing its ancestral area: Early models favored a Gondwanan source, whereas recent analyses support a Laurasian cradle. These differences underscore the need for denser sampling, improved fossil calibration, and more integrative analytical frameworks to resolve the early biogeographic history of Orchidaceae.

### Diversification of Orchidaceae

The species diversity of Orchidaceae exhibits pronounced spatial heterogeneity worldwide, with primary diversification centers and the highest species richness located in Southeast Asia and Neotropics (Givnish et al., 2016; Pérez‐Escobar et al., 2022, 2024). The dispersal and diversification of orchids have been shaped by the combined effects of long‐distance dispersal, orogeny, island isolation, the Cretaceous–Paleogene (K–Pg) boundary event (~66 Ma), sea‐level fluctuations, and recurrent climatic shifts (Givnish et al., 2016; Li et al., 2019b; Gamisch et al., 2021; Zhang et al., 2023a). Concurrently, several key biological traits intrinsic to orchid evolution have significantly facilitated their diversification. These include the production of minute seeds adapted for anemochory (wind dispersal), multiple independent origins of the epiphytic habit, specialized deceptive pollination strategies, and mycorrhizal symbioses (Gravendeel et al., 2004; Tremblay et al., 2005; Freudenstein and Chase, 2015; Givnish et al., 2015). Through synergistic interactions among these geological, climatic, and biological factors, orchid diversity hotspots have predominantly emerged in tropical regions.

**Figure 2:**
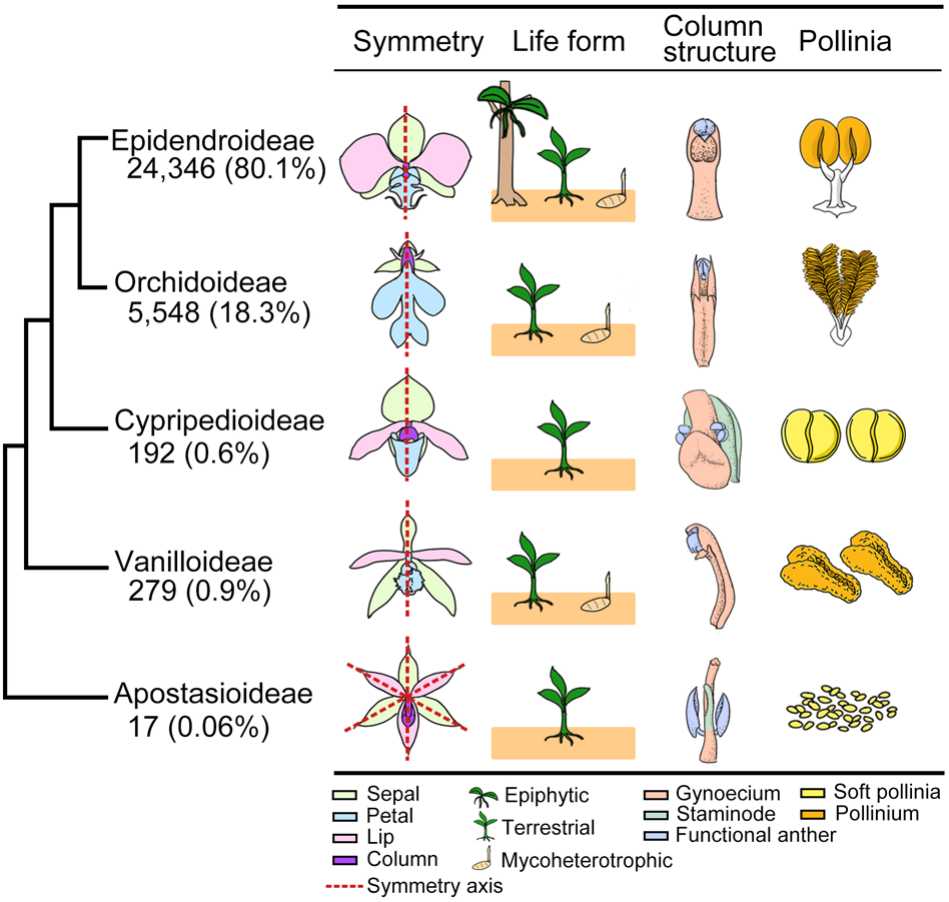
Evolution of key morphological innovations across five Orchidaceae subfamilies: symmetry, life form, column structure, and pollinia Numbers below each subfamily indicate the number of species included in that subfamily and the percentage of Orchidaceae species represented by that subfamily.

Patterns of diversification vary considerably across clades and regions, with the highest speciation rates concentrated in epiphytic lineages. The rapid radiation of Epidendroideae has been attributed to the evolution of epiphytism and key floral innovations linked to pollinator specialization, such as pollinia and highly derived labellar morphologies (Figure 2; Freudenstein and Chase, 2015). The evolution of succulent pseudobulbs and other water‐storage adaptations has been interpreted as key innovations that enabled epiphytic orchids to persist and expand into seasonally dry forests during Eocene cooling events (Collobert et al., 2023). Long‐distance dispersal has likewise been a major evolutionary driver: Phylogenomic analyses estimate at least 74 transoceanic dispersal events during the evolutionary history of the family, fostering the establishment of tribes and genera across continents (Givnish et al., 2016, 2018). Regionally, Southeast Asia has been identified as an important diversification accelerator, whereas the Neotropics provided particularly favorable conditions for the explosive radiation of Epidendroideae. Furthermore, post‐K–Pg climatic perturbations and the expansion of modern tropical rainforests likely amplified diversification rates (Zhang et al., 2023a).

Among regional orchid radiations, the Neotropics provide one of the clearest examples of how geological dynamism and ecological complexity interact to promote diversification. The rapid uplift of the Andes, the emergence of the Isthmus of Panama, recurrent volcanism, and tectonic activity together generated novel habitats and dispersal barriers, triggering an exceptional diversification pulse over the past five million years (Pérez‐Escobar et al., 2017a, 2022, 2024). Importantly, mountains are not barriers to orchid dispersal; instead, they provide complex topographic and ecological gradients that promote rapid radiation. The interplay of epiphytic growth forms, specialized pollination interactions, and environmental heterogeneity has been fundamental in shaping the extraordinary diversity of Neotropical orchids.

At the genus level, diversification histories illustrate strikingly heterogeneous trajectories. *Bulbophyllum*, the largest orchid genus (~2,000 spp.), experienced a Quaternary diversification burst, likely facilitated by long‐distance dispersal from its Asia‐Pacific origin to Madagascar, Africa, and the Neotropics, yielding a pantropical distribution (Gamisch and Comes, 2019). Drivers of this radiation include climatic oscillations during the Last Glacial Maximum, rainforest expansion, island isolation, and the evolution of crassulacean acid metabolism (CAM) photosynthesis (Gamisch et al., 2016, 2021; Hu et al., 2022). In contrast, *Dendrobium*, the third‐largest genus (~1,600 spp.), confined to Asia and Australasia, diversified in association with Cenozoic climatic and geological changes (Xiang et al., 2016). The Australian lineage originated from Asian ancestors through dispersal across Wallace's Line during the Oligocene, coinciding with late Oligocene warming, humidification, and tropical rainforest expansion. Subsequent diversification of the Asian and Australian lineages near the Oligocene–Miocene boundary was likely promoted by climatic cooling, Mi‐1 glaciation, sea‐level fluctuations, and geographic isolation, with additional middle Miocene dispersal events further shaping the current distribution of the genus (Chen et al., 2025). Similarly, the biogeographic history of *Phalaenopsis* reflects glacial–interglacial sea‐level fluctuations that intermittently connected Southeast Asian archipelagos via land bridges (Tsai, 2011; Tsai et al., 2015). In montane Asia, the diversification of *Dendrobium*, *Holcoglossum*, *Pleione*, and *Gastrochilus* has been tightly linked to orogeny (uplift of the Hengduan Mountains and the Qinghai–Tibetan Plateau), intensification of the Asian monsoon, and Pleistocene climatic fluctuations (Fan et al., 2009; Zhao et al., 2020; Li et al., 2022b; Wu et al., 2023; Chen et al., 2025).

The diversification of Cypripedioideae further exemplifies the integration of geological, climatic, and biological drivers. *Paphiopedilum* originated in southern China and mainland Southeast Asia and subsequently dispersed across the Sunda Shelf, with speciation strongly influenced by sea‐level oscillations and regional orogeny (Tsai et al., 2020; Liao et al., 2024; Górniak et al., 2025). *Cypripedium* originated in Central America and expanded to Eurasia during the Eocene–Oligocene transition via intermittent Beringian and North Atlantic land bridges (Liu et al., 2021; Liao et al., 2024). Both genera exhibit diversification tightly coupled with geomorphological dynamics and distinctive pollination biology. In particular, the slipper‐shaped lip facilitates deceptive pollination, a trait considered an important driver of reproductive isolation and speciation (Pemberton, 2013). Similarly, sexual deception in some genera, such as *Ophrys* and *Chiloglottis*, has been identified as a key innovation that accelerates species formation through enhanced prezygotic isolation and gene flow restriction (Breitkopf et al., 2015; Whitehead et al., 2015; Salvado et al., 2025).

In summary, the diversification of Orchidaceae is driven by the synergistic effects of ecological innovations (epiphytism, succulence, pollination strategies), geological processes (orogeny, land‐bridge dynamics, island isolation), climatic oscillations, and repeated long‐distance dispersal. These interacting forces, operating across lineages and regions, have collectively shaped the extraordinary species richness of orchids and cemented their status as one of the largest families of flowering plants.

## GENOMICS AND MORPHOGENESIS OF ORCHIDACEAE

### Advances in genome sequencing of Orchidaceae

Whole‐genome sequencing is a critical resource for elucidating evolutionary mechanisms, offering unprecedented opportunities to explore the origin, adaptive evolution, and molecular basis of hallmark traits in the Orchidaceae. Orchids exhibit remarkable morphological and ecological diversity, driven fundamentally by genomic diversity. Previous studies have revealed significant variation in orchid genome sizes, ranging 168‐fold from 0.33 Gb (*Trichocentrum maduroi*) to 55 Gb (*Pogonia ophioglossoides*) (Leitch et al., 2009). Compared to model plants like *Arabidopsis thaliana* (ca. 0.134 Gb) (Hou et al., 2022), orchid genomes typically exhibit high heterozygosity and abundant repetitive sequences (up to 70% or more; Table 2; Sharma and Mukai, 2015; Ai et al., 2021; Yang et al., 2025). These characteristics pose substantial challenges for sequencing and assembly, including fragmented assemblies, difficulties in resolving repetitive regions, and sequence ambiguities due to heterozygosity (Sun et al., 2022). Overcoming these challenges requires advanced sequencing technologies and assembly strategies to achieve high‐quality chromosome‐level genomes.

**Table 2:** Whole genome sequences of reported orchids species

Subfamily	Species	Sequencing method	Genome size (Mb)	Chr. No. (1*n*)	Contig N50 (Mb)	Scaffold N50 (Mb)	Protein coding genes	Repetitive sequences (%)	Assembly BUSCO (%)	Reference
Apostasioideae	*Apostasia shenzhenica*	Illumina, PacBio	349	34	0.08	3.03	21,841	42.05	93.62	Zhang et al., 2017
	*Apostasia ramifera*	Illumina	365.59	–	0.03	0.29	22,841	44.99	92.30	Zhang et al., 2021c
	*Apostasia fujianica*	Illumina, PacBio, Hi‐C	340.90	35	2.37	9.88	21,724	–	91.80	Lan et al., 2025
Cypripedioideae	*Cypripedium singchii*	Illumina, PacBio, Hi‐C	40,929.10	10	1.17	3,726.49	32,412	–	85.01	Lan et al., 2025
Vanilloideae	*Vanilla planifolia* ‘Daphna’	Illumina, Nanopore, Hi‐C	A: 736.8 B: 744.2	14	0.40	42.00	A: 29,167 B: 29,180	46.2	A: 91.0 B: 89.3	Hasing et al., 2020
	*Vanilla planifolia* CR0040	Illumina, PacBio HiFi, Nanopore	A: 1,500 B: 1,900	14	0.92	1.2	A: 26,392 B: 32,736	72	A: 80.7 B: 79.3	Piet et al., 2022
Orchidoideae	*Anoectochilus roxburghii* ‘Jinkang No.1’	Illumina, PacBio HiFi, Hi‐C	5,165.85	20	24.90	60.99	88,106	76.42	96.00	Fang et al., 2025
	*Anoectochilus roxburghii* ‘Xiaoyuanye’	Illumina, PacBio HiFi, Hi‐C	A: 1,920 B: 1,930	20	A: 22.72 B: 22.17	A: 104.19 B: 105.51	A: 26,239 B: 26,324	A: 76.54 B: 76.68	A: 93.5 B: 92.9	Xu et al., 2025
	*Platanthera zijinensis*	Illumina, PacBio, Hi‐C	4,185.53	21	1.77	192.35	24,513	77.38	88.66	Li et al., 2022a
	*Platanthera guangdongensis*	Illumina, PacBio, Hi‐C	4,197.04	21	1.57	193.14	22,559	82.18	72.06	Li et al., 2022a
	*Ophrys sphegodes*	Illumina, Nanopore, Hi‐C	5,219.75	18	0.75	218	42,549	78	84.9	Russo et al., 2024
Epidendroideae	*Gastrodia elata* 1	Illumina	1,061.09	–	0.07	4.9	18,969	66.18	72.4	Yuan et al., 2018
	*Gastrodia elata* 2	Illumina	1,120	–	0.10	1.64	24,484	68.34	81.85	Chen et al., 2020c
	*Gastrodia elata* 3	Illumina, PacBio, Hi‐C	1,043	18	21.33	–	21,115	66.36	70.80	Xu et al., 2021
	*Gastrodia elata* 4	Illumina, PacBio, Hi‐C	1,008	18	9.18	50.6	18,844	74.92	64.9	Bae et al., 2022
	*Gastrodia menghaiensis*	Illumina, PacBio, Hi‐C	862.84	18	2.37	6.82	14,233	62.57	67.90	Jiang et al., 2022a
	*Bletilla striata*	PacBio HiFi, Hi‐C	A: 2,370 B: 2,430	16	A: 1.65 B: 1.66	A: 146.39 B: 150.22	A: 26,673 B: 26,891	–	A: 97.52 B: 97.24	Jiang et al., 2022b
	*Dendrobium officinale* 1	Illumina, PacBio	1,447	19	0.03	0.08	35,567	63.33	–	Yan et al., 2015
	*Dendrobium catenatum*	Illumina	1,008.55	19	0.03	0.39	28,910	78.1	89.1	Zhang et al., 2016
	*Dendrobium officinale* 2	Illumina, PacBio, Hi‐C	1,230	19	1.44	63.07	27,631	76.77	93.9	Niu et al., 2021
	*Dendrobium huoshanense*	Illumina, PacBio, Hi‐C	1,285	19	0.60	71.79	21,070	79.38	86.6	Han et al., 2020
	*Dendrobium chrysotoxum*	MGISEQ‐2000, PacBio, Hi‐C	1,370	19	1.54	67.80	30,044	62.81	90.30	Zhang et al., 2021b
	*Dendrobium nobile*	Illumina, PacBio, Hi‐C	1,192.38	19	1.62	64.46	29,476	61.07	96.22	Xu et al., 2022
	*Dendrobium thyrsiflorum*	Illumina, PacBio, Hi‐C	1,435.49	20	2.93	72.89	28,702	70.64	97.50	Chen et al., 2024c
	*Dendrobium devonianum*	Illumina, Nanopore, Hi‐C	1,110	19	9.03	56.12	32,759	74.71	96.80	Wang et al., 2025b
	*Dendrobium moniliforme*	Illumina, PacBio, Hi‐C	1,200	19	3.97	–	27,231	71.59	87.3	Yang et al., 2025
	*Dendrobium aphyllum*	Illumina, PacBio HiFi, Hi‐C	1,630.83	19	25.39	100.29	29,611	64.84	92.3	Li et al., 2025a
	*Dendrobium hercoglossum*	Illumina, PacBio HiFi, Hi‐C	1,048.33	19	7.85	63.73	31,254	57.86	96.2	Li et al., 2025a
	*Dendrobium ellipsophyllum*	Illumina, PacBio HiFi, Hi‐C	1,103.11	19	18.16	68.36	22,062	60.13	96.3	Li et al., 2025a
	*Dendrobium formosum*	Illumina, PacBio HiFi, Hi‐C	1,375.86	19	5.71	79.89	24,370	57.48	95.3	Li et al., 2025a
	*Dendrobium lindleyi* 1	Illumina, PacBio HiFi, Hi‐C	948.16	19	20.28	48.10	23,948	53.78	94.8	Li et al., 2025a
	*Dendrobium jenkinsii*	Illumina, PacBio HiFi, Hi‐C	862.47	19	11.48	42.11	23,493	53.01	95.4	Li et al., 2025a
	*Dendrobium leonis*	Illumina, PacBio HiFi, Hi‐C	1,120.37	19	8.73	64.30	26,792	57.56	95.8	Li et al., 2025a
	*Dendrobium crumenatum*	Illumina, PacBio HiFi, Hi‐C	913.92	19	7.51	49.26	26,995	54.50	94.4	Li et al., 2025a
	*Dendrobium smillieae*	Illumina, PacBio HiFi, Hi‐C	1,216.84	19	6.88	71.12	31,440	56.10	95.2	Li et al., 2025a
	*Dendrobium secundum*	Illumina, PacBio HiFi, Hi‐C	1,185.19	19	8.45	63.34	22,160	65.91	96.6	Li et al., 2025a
	*Dendrobium crocatum*	Illumina, PacBio HiFi, Hi‐C	1,113.27	19	25.01	56.07	23,837	59.94	96.6	Li et al., 2025a
	*Dendrobium* ‘Chao Praya Smile’	Illumina, PacBio HiFi, Hi‐C	839.48	19	8.71	43.39	20,004	63.14	96.7	Li et al., 2025a
	*Dendrobium discolor*	Illumina, PacBio HiFi, Hi‐C	745.30	19	19.48	39.08	20,987	61.30	96.9	Li et al., 2025a
	*Dendrobium tetragonum*	Illumina, PacBio HiFi, Hi‐C	977.66	19	23.45	55.27	24,759	61.58	96.6	Li et al., 2025a
	*Dendrobium bullenianum*	Illumina, PacBio, Nanopore, Hi‐C	946.75	19	8.92	48.73	30,393	58.04	95.8	Chen et al., 2025
	*Dendrobium cariniferum*	Illumina, PacBio, Nanopore, Hi‐C	1,382.51	19	4.37	70.57	29,304	56.58	93.6	Chen et al., 2025
	*Dendrobium exile*	Illumina, PacBio, Nanopore, Hi‐C	946.12	19	5.29	49.27	29,426	59.38	96.3	Chen et al., 2025
	*Dendrobium lindleyi* 2	Illumina, PacBio, Nanopore, Hi‐C	943.87	19	4.75	53.69	29,446	57.29	94.3	Chen et al., 2025
	*Dendrobium parcum*	Illumina, PacBio, Nanopore, Hi‐C	1,509.38	19	2.18	97.53	28,952	59.31	97.3	Chen et al., 2025
	*Dendrobium porphyrochilum*	Illumina, PacBio, Nanopore, Hi‐C	1,236.54	20	1.01	76.31	29,211	68.29	93.9	Chen et al., 2025
	*Dendrobium secundum*	Illumina, PacBio, Nanopore, Hi‐C	1,327.81	20	1.36	71.48	29,132	60.9	95.2	Chen et al., 2025
	*Dendrobium spatella*	Illumina, PacBio, Nanopore, Hi‐C	1,123.28	19	2.78	56.84	29,333	55.95	96.8	Chen et al., 2025
	*Phalaenopsis equestris*	Illumina	1,086	19	0.02	0.36	29,431	62	89.1	Cai et al., 2015
	*Phalaenopsis* ‘KHM190’	Illumina	3,104.27	–	0.001	0.10	41,153	–	–	Huang et al., 2016
	*Phalaenopsis aphrodite*	Illumina	1,025.1	19	0.02	19.70	28,902	60.3	~95	Chao et al., 2018
	*Papilionanthe* Miss Joaquim ‘Agnes’	Illumina, Nanopore, Hi‐C	2,570	19	–	159.5	31,529	78	95.6	Lim et al., 2022
	*Gongora gibba*	Illumina, PacBio, Hi‐C	1,831	–	0.38	1.7	17,374	82.95	86.6	Guizar Amador et al., 2024
	*Angraecum sesquipedale*	Illumina, PacBio, Hi‐C	2,103.17	19	1.55	105.75	25,985	69.13	93.50	Jiang et al., 2025
	*Cymbidium sinense*	Illumina, Nanopore, Hi‐C	3,525.14	20	1.11	–	29,638	77.78	91.0	Yang et al., 2021
	*Cymbidium goeringii* 1	Illumina, PacBio, Hi‐C	4,068.80	20	1.04	209.04	30,897	77.65	86.90	Sun et al., 2021
	*Cymbidium goeringii* 2	Illumina, PacBio, Hi‐C	3,990.52	20	0.33	178.2	29,556	88.87	87.8	Chung et al., 2022
	*Cymbidium ensifolium*	Illumina, PacBio, Hi‐C	3,620.01	20	1.21	154.88	29,073	80.58	87.00	Ai et al., 2021
	*Cymbidium mannii*	MGISEQ‐2000, Illumina, PacBio HiFi, Nanopore, Hi‐C	2,880	20	22.70	130.73	27,192	82.8	97.0	Fan et al., 2023
	*Cymbidium tracyanum*	Illumina, PacBio, Hi‐C	3,793.64	20	1.66	161.69	42,249	88.57	–	Tu et al., 2025
	*Cremastra appendiculata*	Illumina, PacBio, Hi‐C	2,058	24	0.68	80.16	20,991	59.15	86.4	Wang et al., 2022

A, haplotype‐A, B, haplotype‐B; – indicates that no data are available.

*Anoectochilus roxburghii ‘Jinkang No.1’ reported by Fang et al. (2025) was sequenced from an autotetraploid individual; the reported genome size of 5.17 Gb refers to the total assembled genome spanning 80 pseudo‐chromosomes.

Advances in sequencing technologies have been pivotal in driving the rapid progress of Orchidaceae genome research, with significant improvements in efficiency and quality from Sanger sequencing to next‐generation sequencing (NGS) and third‐generation sequencing. Traditional Sanger sequencing, used for early model plants and crop genomes (The Arabidopsis Genome Initiative, 2000; Yu et al., 2002), was time‐consuming, costly, and unsuitable for complex orchid genomes. NGS technologies, such as Roche 454 and Illumina platforms, dramatically increased throughput and reduced costs, enabling large‐scale genome sequencing. However, the short read lengths (100–300 bp) generated by NGS struggle to span long repetitive regions in orchid genomes, resulting in highly fragmented assemblies with numerous unfilled gaps and unplaced scaffolds. Consequently, early orchid genomes were mostly draft assemblies (Cai et al., 2015; Yan et al., 2015; Zhang et al., 2017).

The advent of third‐generation sequencing technologies has provided a breakthrough. Pacific Biosciences (PacBio) and Oxford Nanopore Technologies (ONT) generate long reads (10–100 kb or longer), effectively spanning complex repetitive regions and improving assembly contiguity. Additionally, chromosome conformation capture (High‐throughput Chromosome Conformation Capture, Hi‐C) and BioNano optical mapping leverage chromatin proximity or physical positioning to facilitate high‐quality chromosome‐level assemblies in orchids (Hasing et al., 2020; Niu et al., 2021; Chung et al., 2022; Li et al., 2022a; Tu et al., 2025). Early third‐generation sequencing had high error rates (ca. 5%–15%), requiring correction with NGS short reads. Recent innovations, such as PacBio High‐Fidelity (HiFi) and ONT R10, combine long reads with high accuracy, significantly enhancing assembly quality and enabling telomere‐to‐telomere (T2T) genome assemblies (Garg et al., 2024). While T2T assemblies have been achieved in model plants and crops (Song et al., 2021; Chen et al., 2023; Zhang et al., 2023b), no such reports exist for Orchidaceae to date. Continued reductions in sequencing costs and advancements in assembly algorithms are poised to accelerate the completion of high‐quality orchid genomes, paving the way for functional and comparative genomics studies.

**Figure 3:**
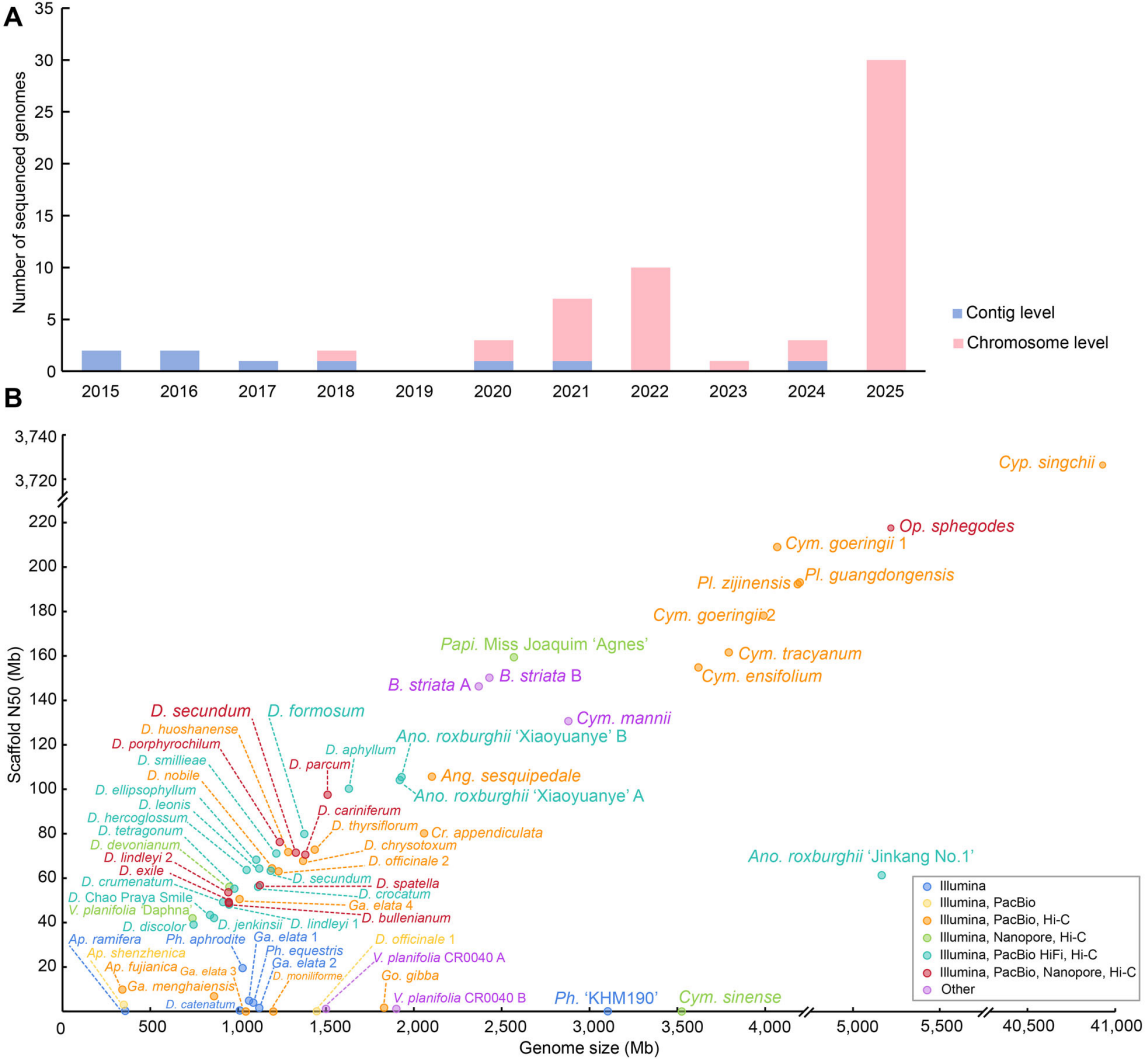
Progress in Orchidaceae genome sequencing **(A)** Number of orchid genomes sequenced at the chromosome level and contig level since the first published Orchidaceae genome. **(B)** Summary of Orchidaceae genome assemblies. The x‐axis represents genome size and the y‐axis indicates scaffold N50. Different colors correspond to different sequencing platforms. “Other” includes combined sequencing strategies such as Illumina + PacBio HiFi + Nanopore, Illumina + PacBio + Nanopore + Hi‐C, PacBio HiFi + Hi‐C, and Illumina + PacBio HiFi + Nanopore + Hi‐C. Scaffold N50 values for *Ga. elata* 3, *D. moniliforme*, and *Cym. sinense* were not reported in the original publications and should not be interpreted as 0 Mb. *Anoectochilus roxburghii* “Jinkang No.1”, reported by Fang et al. (2025), was sequenced from an autotetraploid individual; the reported genome size of 5.17 Gb refers to the total assembly spanning 80 pseudochromosomes.

In 2015, the whole‐genome sequence of *Phalaenopsis equestris* marked a milestone as the first fully sequenced and analyzed genome of an Orchidaceae species and a CAM plant (Cai et al., 2015). Subsequently, orchid genome sequencing progressed steadily, with a significant acceleration in 2025 (Figure 3A). To date, 61 Orchidaceae genomes spanned all five subfamilies (Apostasioideae, Cypripedioideae, Vanilloideae, Orchidoideae, Epidendroideae), covering 15 genera and 54 species (including 7 cultivars; Table 2). Sequenced genera included *Apostasia*, *Cypripedium*, *Vanilla*, *Anoectochilus*, *Platanthera*, *Ophrys*, *Gastrodia*, *Phalaenopsis*, *Papilionanthe*, *Dendrobium*, *Bletilla*, *Cymbidium*, *Cremastra*, *Gongora*, and *Angraecum*. Among these, genomic data were predominantly concentrated in Epidendroideae, with *Dendrobium* accounting for over half of all available genomes.

Orchid genomes display remarkable levels of diversity. A striking variation is observed in genome size, which spans from 0.34 Gb in *Apostasia fujianica* to a staggering 43.19 Gb in *Cypripedium singchii*, representing a difference exceeding 120‐fold (Figure 3B; Table 2). The chromosome number (1 C) ranges from 10 (*Cyp. singchii*) to 35 (*Ap. fujianica*) (Lan et al., 2025). Sequencing and assembly strategies have evolved from early short‐read approaches (e.g., Illumina) to chromosome‐scale, high‐quality assemblies integrating long‐read technologies (PacBio, Oxford Nanopore) with chromatin conformation capture (Hi‐C; Figure 3B). This advancement has elevated contig N50 lengths from 0.02 Mb to over 20 Mb (Table 2; Cai et al., 2015; Chao et al., 2018; Li et al., 2025a). For instance, the genome assembly of *Dendrobium officinale* improved contig N50 from 0.03 Mb to 1.44 Mb, with scaffold N50 reaching 63.07 Mb (Figure 3B; Table 2; Yan et al., 2015; Zhang et al., 2016; Niu et al., 2021). The number of annotated protein‐coding genes varies considerably across orchid species, ranging from 14,233 (*Gastrodia menghaiensis*) to 42,249 (*Cymbidium tracyanum*) (Jiang et al., 2022a; Tu et al., 2025). As a fully mycoheterotrophic lineage, *Gastrodia* spp. display significantly reduced gene counts (ca. 14,000–21,000), attributable to gene losses associated with their specialized heterotrophic lifestyle (Yuan et al., 2018; Jiang et al., 2022a).

Repetitive sequences, constituting over 60% of the genome in the vast majority of orchids, are a key determinant underlying the extensive genome size variation. Notably, the high proportion of repetitive sequences (exceeding 77%) in *Cymbidium*, *Platanthera*, and *Ophrys sphegodes* contributed to their large genome sizes of over 3 Gb (Ai et al., 2021; Sun et al., 2021; Yang et al., 2021; Li et al., 2022a; Russo et al., 2024; Tu et al., 2025), whereas the relatively low repetitive content (under 45%) in *Apostasia* was associated with its compact genome of about 350 Mb (Table 2; Zhang et al., 2017, 2021c). Repetitive sequences have also been increasingly recognized as important contributors to the emergence and diversification of key plant traits. Previous studies indicated that TE insertions can alter gene copy number, disrupt or create cis‐regulatory elements, modify chromatin and DNA methylation states, and generate structural variation, thereby influencing the expression of nearby genes (Tao et al., 2025). However, current evidence linking TEs to orchid innovations remains largely indirect, despite the prominence of repeat‐rich genomes in many orchids. Future studies should pay more attention to how repeat accumulation affects genome evolution, gene regulation, and phenotypic diversification in orchids, rather than only comparing repeat content among species. This will help clarify whether repetitive sequences have contributed to the evolution of key orchid innovations.

Through comparative genomics, population genomics, evolutionary analyses, and transcriptomics, researchers can reconstruct the ancestral gene toolkit of orchids, elucidating the origins of novel gene families, gene family expansion and contraction, and the molecular mechanisms underlying unique adaptive traits. These high‐quality genomes provide a robust foundation for unraveling the diversity and evolutionary history of Orchidaceae.

### Genomic insights into the origin and early diversification of Orchidaceae

Phylogenomic analyses based on conserved single‐copy genes confirm that Orchidaceae originated from a common ancestor and underwent subsequent radiation, with the subfamily Apostasioideae representing the basal lineage. The genome of *Apostasia* (e.g., *Ap. shenzhenica*) provides a critical reference for inferring the genomic structure and gene content of the most recent common ancestor of extant orchids (Zhang et al., 2017). By integrating single‐copy genes derived from whole genomic and transcriptomic datasets, Zhang et al. (2017) reconstructed a phylogenetic tree encompassing 12 orchid species and estimated the crown age of Orchidaceae, corresponding to the divergence of Apostasioideae, to be approximately 93 Ma. Subsequent analyses using whole‐genome data estimated the origin of the family to be between ca. 80–110 Ma, which is consistent with estimates based on plastid and transcriptomic data, and further supported the established phylogenetic relationships among the five major subfamilies. Hasing et al. (2020) estimated that *Vanilla* diverged from the lineage leading to *Phalaenopsis* and *Dendrobium* around 77 Ma. In a more recent study, Li et al., (2022a) inferred the divergence between Epidendroideae and Orchidoideae to have occurred approximately 60 Ma. Moreover, based on fossil‐calibrated phylogenies of 17 *Dendrobium* species, Yan et al. (2015) estimated that the divergence between Asian and Australian clades within *Dendrobium* occurred around 28.1 Ma. It is noteworthy that variations among these estimates may arise from differences in taxon sampling, gene sets used, and analytical methods, highlighting the need for further studies with expanded genomic and taxonomic representation.

Whole‐genome duplication (WGD) events have played a crucial role in the origin and evolution of the Orchidaceae family. Through comparative genomics and analysis of synonymous substitution rates (*K*_s_), the genome study of *Ph. equestris* first identified a unique ancient polyploidization event specific to orchids, which is hypothesized to have occurred prior to the divergence of most orchid subfamilies (Cai et al., 2015). Further analysis of the genome of *Ap. shenzhenica* (Zhang et al., 2017) revealed that orchids have undergone two WGD events: The earlier one, the τ WGD, was shared among most monocotyledonous plants, while the more recent one, termed the orchid‐specific α° WGD, was specific to orchids and predates the divergence of the five subfamilies. Subsequent genomic studies, including those of *Vanilla planifolia* (Hasing et al., 2020; Piet et al., 2022), *Cym. ensifolium* (Ai et al., 2021), and the *Dendrobium* genus (Niu et al., 2021; Li et al., 2025a), have not identified additional orchid‐specific WGD events, suggesting that the evolutionary trajectory of orchids primarily relied on the genetic foundation provided by these two WGD events.

Multiple studies have highlighted that WGD events are not randomly distributed over time but are strongly associated with periods of global environmental upheaval or mass extinction (Silva et al., 2021; Van de Peer et al., 2021). The orchid‐specific α° WGD has been estimated to date to the K–Pg boundary (Hasing et al., 2020; Zhang et al., 2017, 2021b), aligning closely with previous estimates of orchid divergence time. It is hypothesized that the WGD event may have provided essential genetic buffering that facilitated the survival of orchids during this crisis. The extensive genomic redundancy generated by WGD supplied abundant raw genetic material, which, through neofunctionalization and subfunctionalization following the K–Pg mass extinction, contributed significantly to the explosive species diversification. This enhanced the adaptive capacity of orchids and underpinned their evolutionary radiation in response to subsequent environmental changes.

Following the orchid‐specific WGD, the five major subfamilies of Orchidaceae underwent rapid diversification within a relatively short timeframe. Genomic analyses reveal that this radiation was not a simple linear process but was driven by distinct patterns of gene loss and expansion unique to each subfamily. Intriguingly, compared to the phylogenetically basal Apostasioideae, which occupies the earliest‐diverging position in the orchid tree of life, the subfamily experienced the most extensive gene loss after WGD (Zhang et al., 2017). In contrast, the more derived Epidendroideae retained the highest number of genes (Li et al., 2025a). This suggests that orchid evolution involved not merely the acquisition of genetic innovations, but also a dynamic process of both gene gain and loss. The primitive morphology of Apostasioideae may not be due to a lack of genes responsible for orchid‐specific traits, but rather to the lineage‐specific loss of certain key genes following WGD (Zhang et al., 2017). Genome evolutionary analyses of *Cym. sinense* revealed substantial expansion of the MADS‐box gene family, accompanied by pronounced expression divergence among paralogs, indicative of potential subfunctionalization or neofunctionalization (Yang et al., 2021). Several key flowering regulators, including *APETALA1* (*AP1*)*/FRUITFULL* (*FUL*) and *SUPPRESSOR OF OVEREXPRESSION OF CONSTANS 1* (*SOC1*), exhibited lineage‐specific expansion patterns that were temporally consistent with the shared Orchidaceae WGD and gene duplication events (Yang et al., 2021). These results suggest that WGD‐derived gene retention and diversification contributed to increased complexity of the flowering regulatory network, thereby providing a genomic basis for floral trait diversification, phenological modulation, and ecological adaptation in orchids.

Chromosome evolution and structural variation likely acted to convert latent genomic plasticity into realized lineage divergence. Orchid karyotypes are highly dynamic, with reported chromosome numbers ranging from 2*n* = 10 to 240 and a common concentration around 2*n* = 38/40 (Sharma and Mukai, 2015). Basic chromosome numbers and karyotypic configurations vary substantially among lineages, consistent with recurrent chromosomal remodeling following polyploidization (Sharma and Mukai, 2015; Baranow et al., 2022). Such remodeling can now be assessed directly at the genome scale. In the artificial hybrid cultivar *D*. “Chao Praya Smile”, 14,588 structural variants (SVs) were identified between the two haplotypes, indicating extensive inter‐haplotype structural divergence within the hybrid genome (Li et al., 2025a). More broadly, pangeneric analyses of 24 chromosome‐level genomes showed that diversification in *Dendrobium* has not been shaped solely by gradual sequence evolution, but also by frequent chromosomal rearrangements and changes in chromosomal state. The prevalent karyotype in the genus is 2*n* = 38, whereas the 2*n* = 40 condition appears to have evolved independently at least twice, implicating recurrent chromosome breakage, fusion, and related rearrangements during genus evolution (Chen et al., 2025). In addition, 3,117 inversions and 3,524 translocations, together with abundant SNPs and InDels, were detected across the genus (Chen et al., 2025). Notably, SV hotspots were enriched for genes associated with phenylpropanoid biosynthesis and plant–pathogen interactions, suggesting that structurally dynamic genomic regions may have contributed to regulatory divergence and environmental adaptation (Chen et al., 2025). Collectively, these findings support that chromosome‐number change and structural variation are important components of the genomic processes underlying lineage differentiation and ecological diversification in orchids.

Transposable elements (TEs), referred to as “jumping genes”, significantly influence genome size and evolution through various mechanisms such as gene duplication and creation, modulation of gene expression, and reorganization of chromosomal structures (Oliver et al., 2013; Xu et al., 2017; Dubin et al., 2018). These processes are likely to facilitate lineage‐specific diversification and adaptive evolution (Puttick et al., 2015). In many orchid genome assemblies, TE‐derived sequences account for approximately 50%–80%, with long terminal repeat (LTR) retrotransposons being the predominant subtypes. At present, the evolutionary dynamics of TE bursts have not been investigated at the scale of Orchidaceae as a whole. Available evidence is limited to a few representative lineages, where TE proliferation histories appear to show lineage‐specific patterns. In *Ophrys sphegodes*, repetitive elements occupy 78% of the genome, with LTR elements alone accounting for 75% (Russo et al., 2024). LTR insertion‐age analysis showed that TE activity in the *Ophrys sphegodes* group began to increase around 3 Ma and peaked at 1.3–0.8 Ma. It coincided with both rapid lineage radiation and glacial–interglacial climatic oscillations in the Mediterranean Basin, implying a potential link between environmental change and TE proliferation. In this context, Russo et al. (2024) proposed that TE expansion may have provided the genomic substrate for metabolic innovation associated with chemical mimicry and pollinator adaptation, thereby facilitating niche specialization and adaptive radiation in this lineage.

A comparable but not identical pattern has been reported in *Dendrobium*. At the genus level, pangeneric analyses of 17 *Dendrobium* genomes showed that genome size variation was largely attributable to TE expansion, with LTR retrotransposons representing the predominant TE class and Copia and Gypsy elements having predominantly amplified over 5 Ma after species divergence (Li et al., 2025a). This expansion was hypothesized to have occurred after the rapid uplift of the Qinghai–Tibet Plateau and associated climatic change, suggesting a possible link between TE activity and regional environmental shifts (Niu et al., 2021). Notably, TE dynamics were not uniform across the genus: The Northern clade harbored a markedly higher proportion of LTR retrotransposons than the Southern clade (44% vs 25%), indicating a clade‐structured pattern of recent TE expansion (Li et al., 2025a). Furthermore, super‐panTE analysis revealed extensive variation in TE composition among species, with unique TEs being associated with stronger suppression of gene expression, higher evolutionary rates, and greater functional divergence than core TEs. TEs have also contributed to transposed gene duplication and the species‐specific expansion of TE‐derived *FAR‐RED IMPAIRED RESPONSE1* (*FAR1*) genes, which may facilitate the adaptation of epiphytic *Dendrobium* orchids to the low red/far‐red light conditions characteristic of forest canopy habitats, thereby promoting ecological adaptation and diversification within the genus (Li et al., 2025a). Thus, the limited evidence currently available suggests that bursts of TE activity may promote gene duplication and genome expansion in orchids and temporally coincide with geographic events and environmental change, thereby potentially contributing to diversification.

Based on current genomic evidence, we propose that orchid genome evolution was shaped by the interaction between intrinsic genomic processes and external Earth‐history events. The orchid‐specific WGD, which has been hypothesized to coincide with the Cretaceous–Paleogene boundary, may have provided genetic buffering and evolutionary potential that facilitated orchid survival and subsequent diversification during a period of severe environmental disturbance. Later geological events and climatic oscillations may have imposed strong ecological selection pressures, promoting lineage‐specific TE bursts, genome restructuring, structural variation, and shifts in gene expression patterns. These processes may have contributed to rapid environmental adaptation and the divergence of pollination‐related traits in different orchid lineages. In contrast, extensive gene loss in mycoheterotrophic orchids suggests that genome reduction also represents an important mechanism underlying the evolution of specialized nutritional strategies. Under this framework, CAM photosynthesis, mycorrhizal dependence, epiphytic adaptation, and floral morphogenesis can be viewed as interconnected outcomes of orchid genome plasticity, linking Earth‐history events with ecological, nutritional, and reproductive innovation.

### Evolution of life forms

#### Evolutionary dynamics of the epiphytic lifestyle in Orchidaceae

**Figure 4:**
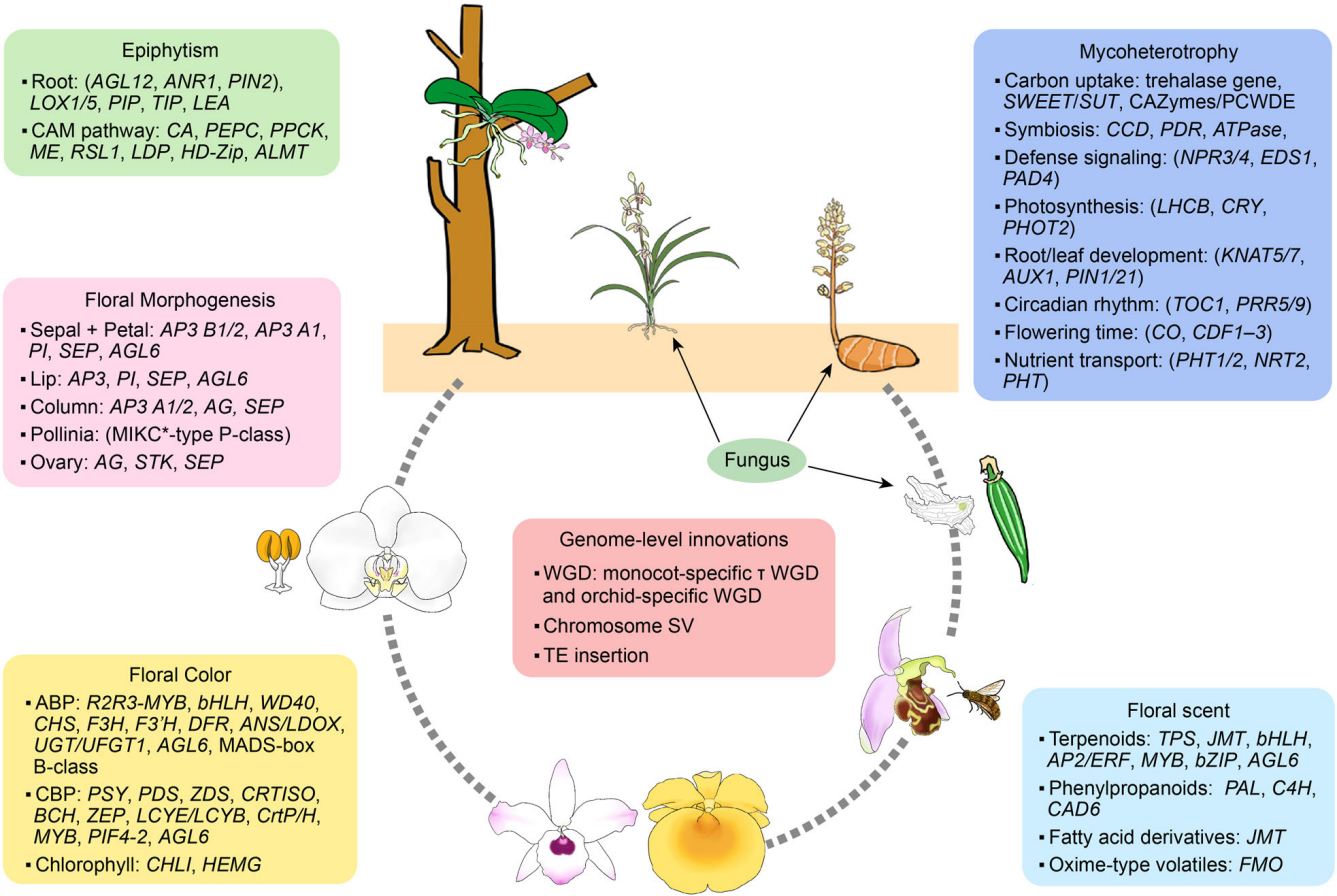
Genome‐level innovations and trait‐associated genes underlying orchid evolutionary innovations Central genome‐level innovations, including monocot‐specific τ whole‐genome duplication (WGD), orchid‐specific WGD, chromosomal structural variation (SV), and transposable element (TE) insertions, may have provided genomic substrates for the evolution of diverse orchid traits. The surrounding panels represent major orchid innovations. Fungi provide nutrients required for orchid seed germination and for the survival of fully and partially mycoheterotrophic species. The transition toward mycoheterotrophy is accompanied by extensive genomic and metabolic remodeling, including the loss or contraction of genes associated with photosynthesis, chlorophyll biosynthesis, circadian regulation, nutrient transport, and light signaling, together with the expansion or retention of genes involved in fungal interaction, nutrient transport, and carbohydrate metabolism. The evolution of epiphytism is mainly associated with the development of aerial roots and the acquisition or enhancement of crassulacean acid metabolism (CAM). Floral innovations include diversification in floral morphogenesis, pigmentation, and scent. Boxes summarize representative genes and gene families associated with these traits. Genes shown in parentheses indicate gene loss or gene family contraction. ABP, anthocyanin biosynthesis pathway; CBP, carotenoid biosynthesis pathway.

Approximately 70% of Orchidaceae species are epiphytes, which enables them to thrive in soil‐scarce environments, thereby expanding their ecological niches (Zhang et al., 2023a). Genomic studies have revealed that the evolution of epiphytic habits is closely associated with specific gene losses and contractions (Figure 4). Comparative genomic analyses between *Apostasia* and epiphytic orchids indicated that root specialization in orchids was linked to the loss of the *AGAMOUS‐LIKE12* (*AGL12*) gene and contraction of the *ARABIDOPSIS NITRATE REGULATED 1* (*ANR1*) gene (Zhang et al., 2017). In the pangeneric genome study of *Dendrobium*, a representative epiphytic orchid lineage, Li et al. (2025a) reported the conserved loss of *AGL12* in the Asian clade, whereas *AGL12* was retained in some Australian species. Chen et al. (2025) reinterpreted this pattern, suggesting that the common ancestor of the Asian and Australian lineages had lost *AGL12*‐like genes, and that the *AGL12*‐like genes detected in Australian species may have been regained with neofunctionalization. Epiphytic orchids also exhibited an expansion of gene families, including *terpene synthase* (*TPS*), pathogen resistant‐related genes, and *lipoxygenase 1* (*LOX1*)*/LOX5* homologs (Zhang et al., 2021c). The genome of *Phalaenopsis* species showed retentions or expansions of genes related to water stress, light response, dehydration‐responsive proteins, secondary metabolic synthases, and stress‐associated transporters (Cai et al., 2015; Chao et al., 2018).

The adventitious roots of epiphytic orchids, critical for their survival, are closely tied to auxin‐related genes (Tian et al., 2022; Sun et al., 2024). These roots are unaffected by gravity, enabling omnidirectional growth and attachment to bark surfaces, a trait correlated with the absence of starch granules/statolith sedimentation and the lack of the auxin efflux carrier *PINFORMED 2* (*PIN2*) gene (Chen et al., 2024b). Epiphytic orchid roots are not only specialized for anchorage, but also play central roles in water acquisition, temporary storage, and desiccation resistance under highly fluctuating canopy environments. The rapid uptake of transient water inputs and the maintenance of internal water balance are likely supported by aquaporin family members, particularly plasma membrane intrinsic protein (PIP) and tonoplast intrinsic protein (TIP), which are regarded as key candidates for transmembrane water transport in orchids (Zheng et al., 2025). Beyond water uptake itself, drought tolerance in epiphytic orchids involves coordinated stress‐response networks, commonly including abscisic acid (ABA) signaling, reactive oxygen species (ROS) scavenging, and broader metabolic reprogramming (Huang et al., 2023). In this context, dehydration‐responsive proteins such as late embryogenesis abundant (LEA) and dehydrin family members are thought to enhance cellular desiccation tolerance by stabilizing membranes and proteins, a role supported by stress‐inducible expression patterns and heterologous expression studies (He et al., 2021; Huang et al., 2023). These genetic adaptations enable epiphytic orchids to develop specialized roots capable of absorbing atmospheric moisture, ensuring survival and reproduction in epiphytic habitats.

The evolution of CAM is strongly associated with the epiphytic lifestyle and represents a key adaptive innovation for occupying arid canopy niches. The orchid‐specific WGD event may have facilitated the evolution of CAM photosynthesis (Cai et al., 2015). In *Ph. equestris*, CAM‐related genes have undergone lineage‐specific duplications and losses, including carbonic anhydrase (*CA*), phosphoenolpyruvate carboxylase (*PEPC*), phosphoenolpyruvate carboxykinase (*PPCK*), and malic enzyme (*ME*). Based on genomic analysis of *Cym. mannii*, Fan et al. (2023) constructed a model of rhythmic metabolite accumulation, highlighting diurnal oscillations in CAM‐related metabolites. Diurnal expression of core CAM genes, particularly *βCA* and *PPC*, suggests their role in temporal carbon fixation. The *PEPC* gene family primarily catalyzes the reaction of HCO_3_^−^ and phosphoenolpyruvate (PEP) to produce oxaloacetate (OAA) and malate (Borland et al., 2009; Deng et al., 2016). Li et al. (2024a) identified 33 *PEPC* genes in orchid genomes, with cis‐regulatory elements in their promoter regions linked to light responsiveness and circadian rhythms observed in both *PEPC‐i* and *PEPC‐ii* genes. Furthermore, studies have identified key regulatory genes associated with carbon fixation, circadian clock regulation, and stomatal movement within the CAM pathway, including *RING finger of seed longevity 1* (*RSL1*) (Heyduk et al., 2019), light‐dependent period (LDP) iron‐sulfur protein (Zhang et al., 2019), members of the homeodomain‐leucine zipper (*HD‐Zip*) family (Huang et al., 2021), and aluminum‐activated malate transporter (*ALMT*) (Peng et al., 2024). The epiphytic lifestyle in Orchidaceae is underpinned by genomic adaptations that facilitate specialized root development, water retention, and CAM photosynthesis, enabling these plants to thrive in challenging, arid environments.

#### Nutrient acquisition in mycoheterotrophic orchids

Most plants obtain energy through photosynthesis by synthesizing carbohydrates, while certain lineages have evolved the ability to acquire carbohydrates from other organisms, a strategy known as heterotrophy. Over 30,000 plant species engage in fungal heterotrophy, among which 880 are fully mycoheterotrophic, with Orchidaceae being a major group exhibiting this trait (Li et al., 2022a). Due to the absence of endosperm in orchid seeds, germination and early seedling development rely entirely on carbon and nutrient supply by specific symbiotic orchid mycorrhizal fungi (sgOMFs), a dependency that may persist into adulthood. The sgOMFs (e.g., *Rhizoctonia* spp.) colonize orchid tissues, forming intracellular hyphal coils known as pelotons, which facilitate carbon transfer (Dearnaley et al., 2016). Fungal‐derived carbon, primarily in the form of trehalose and glucose, is delivered via two mechanisms: direct uptake through active hyphae or release upon peloton degradation (Zhao et al., 2024b). Carbon translocation involves energy‐independent transporters, including Sugars Will Eventually be Exported Transporters (SWEETs) and H^+^‐hexose symporters (Zhao et al., 2024b). Genes encoding carbohydrate‐active enzymes (CAZymes), including plant cell wall‐degrading enzymes (PCWDEs) (Figure 4), may facilitate fungal penetration and colonization during symbiotic germination by modifying host cell walls and influencing host immune responses (Kohler et al., 2015; Chen et al., 2022; De Rose et al., 2024). Phytohormones also play regulatory roles; for instance, gibberellins (GAs) contribute to orchid seed germination by de‐repressing mycorrhizal signaling pathways, thereby avoiding negative effects on fungal colonization. This contrasts with arbuscular mycorrhizal symbiosis, where GAs typically suppress fungal development, highlighting how orchids have repurposed GA‐mediated regulation into a germination strategy (Miura et al., 2024).

*Gastrodia elata* is widely regarded as a model system for studying obligate mycoheterotrophy in orchids. Yuan et al. (2018) reported massive gene family contractions in its genome, including the loss of key photosynthesis‐related genes (e.g., chlorophyll synthesis and Rubisco). In contrast, gene families involved in fungal cell wall degradation, such as glycoside hydrolases and ureases, expanded, facilitating nutrient absorption from fungal hyphae. Additionally, strigolactone signaling (e.g., carotenoid cleavage dioxygenases (CCDs) and ABC transporters, PDRs) and ATPase genes showed notable expansion, likely supporting altered energy metabolism. Yuan et al. (2018) proposed that genomic plasticity underlies the adaptation to heterotrophy in orchids. Similarly, Xu et al. (2021) assembled the *Ga. elata* genome and documented losses not only in photosynthetic genes but also in circadian clock regulators (e.g., *TIMING OF CAB EXPRESSION 1* (*TOC1*), *PSEUDO‐RESPONSE REGULATOR 5* (*PRR5*), *PRR9*), flowering time controllers (e.g., *CONSTANS* (*CO*), *CYCLING DOF FACTOR1–3* (*CDF1–3*)), immune components (e.g., nonexpressor of pathogenesis‐related 3/4 (*NPR3/4*), *ENHANCED DISEASE SUSCEPTIBILITY 1* (*EDS1*), phytoalexin deficient 4 (*PAD4*)), nutrient transporters (e.g., nitrate transporter 2 (*NRT2*), phosphate transporter (*PHT*)), and root/leaf development genes (e.g., *KNOTTED1‐like homeobox 5/7* (*KNAT5/7)*, *AUXIN RESISTANT 1* (*AUX1*), *PIN21*). These losses reflect a “reduced genome” strategy adapted to dark forest understories. Comparative genomic analyses across trophic types, including initial mycoheterotrophy, parasitism, and autotrophy, revealed convergent gene loss patterns between *Ga. elata* and fully parasitic plants such as *Cuscuta australis*, underscoring gene loss as a key evolutionary mechanism in the adaptation to heterotrophy. More recently, Yuan et al. (2025) demonstrated that *Ga. elata* has lost key nitrogen assimilation genes and indole‐3‐acetic acid (IAA) biosynthesis genes, while its symbiotic fungus *Mycena purpureofusca* retains these functions, compensating for host deficiencies. Furthermore, Hua et al. (2024) elucidated the carbon acquisition mechanism: *Ga. elata* employs strigolactones to regulate ROS homeostasis in *Armillaria* spp., primarily via a novel aquaporin gene *AgAQPA* (belonging to the ‘other aquaglyceroporin’ subfamily), which transports hydrogen peroxide (H_2_O_2_) across membranes to attract the fungus and facilitate carbon transfer. Collectively, these studies reveal that *Ga. elata*, as an obligately mycoheterotrophic orchid, has achieved high specialization toward a fully fungus‐dependent lifestyle through a series of evolutionary adaptations, including gene loss, metabolic reprogramming, and hormone‐fungal interactions.

Partially mycoheterotrophic (mixotrophic) orchids offer critical insights into transitional states along the continuum toward full heterotrophy. Studies have revealed that some green‐leaved orchids supplement their carbon intake via fungal partners under low‐light conditions, as observed in *Liparis* (Gebauer and Meyer, 2003), *Corallorhiza* (Kim et al., 2015), *Cypripedium* and *Cephalanthera* (Preiss et al., 2010). Li et al., (2022a) conducted a comparative genomic analysis of two sister species with distinct trophic strategies: The partially mycoheterotrophic *Platanthera zijinensis* (with leaves) and the fully mycoheterotrophic *P. guangdongensis* (lacking both leaves and roots). The genome of the fully heterotrophic species showed extensive gene losses, including genes related to photosynthesis (e.g., light‐harvesting chlorophyll A/B binding (*LHCB*)), photoreceptors (e.g., cryptochromes (*CRY*), *phototropins2* (*PHOT2*)), and auxin transporters (*PIN1*), reflecting adaptations to dark environments. Concurrent reductions in nitrogen and phosphorus transporters (e.g., *NRT2*, *PHT1/2*) suggest increased reliance on fungal‐derived nutrients such as NH_4_^+^, glutamine, and phosphorus, indicating a shift toward a parasitic relationship. Notably, the expansion of trehalase genes (2–4 copies) in most orchids supports the hijacking of fungal trehalose. This process primarily involves high expression of trehalase, which hydrolyzes trehalose into glucose. The glucose is subsequently converted to sucrose via sucrose synthase, and then distributed to various tissues through sucrose transporter (SUT) proteins, thereby ensuring energy supply and perpetuating the nutrient acquisition strategy characteristic of the protocorm stage. The increased *K*_S_ suggests an accelerated evolutionary pace in fully heterotrophic lineages, likely due to relaxed purifying selection. This evidence aligns with the “Waiting Room Hypothesis” (Selosse et al., 2009, 2022), which posits that plants initially engage in loose fungal associations. Environmental pressures and genomic adjustments then drive increasingly dependent relationships, culminating in full mycoheterotrophy.

### Reproductive organs driving diversification in Orchidaceae

#### Floral development and morphogenesis

The remarkable floral morphological diversity in orchids (Orchidaceae) may have been facilitated by an orchid‐specific WGD event in their common ancestor, which provided a rich genetic toolkit for functional diversification. The MADS‐box gene family, a key regulator of floral organ development, controls processes such as flowering time, floral organ identity, as well as fruit and vegetative development, playing a central role in the evolution of orchid floral morphology. Genomic studies have revealed that expansions and contractions of MADS‐box genes underlie the highly specialized morphologies in orchids. For instance, the expansions of class C/D, B‐class *AP3*, and *AGL6* genes contributed to floral organ specialization (Cai et al., 2015). Particularly, paleo*AP3* genes were further subdivided into four subclades and were thought to have diversified through subfunctionalization and/or neofunctionalization. Together with changes in their interaction partners among other MADS‐box proteins, this diversification may enable organ‐specific rewiring of the perianth regulatory program (e.g., lip versus lateral tepals) while retaining the core floral developmental framework (Tsai et al., 2014). In *Apostasia*, the loss of *AP3* and E‐class genes resulted in an undifferentiated lip and partially fused gynostemium, leading to a reversion from bilateral to radial symmetry (Zhang et al., 2017). The loss of MIKC*‐type P‐class genes likely facilitated pollinium formation, enhancing pollination efficiency and reproductive isolation. In *Dendrobium*, MADS‐box gene expansion has been linked to the genus's remarkable structural diversity (Zhang et al., 2016, 2024b). In *Cymbidium*, the number of MADS‐box genes reached 69–71 with notable expansion in type II subfamilies, supporting unique floral developmental models (Ai et al., 2021; Sun et al., 2021; Yang et al., 2021).

**Figure 5:**
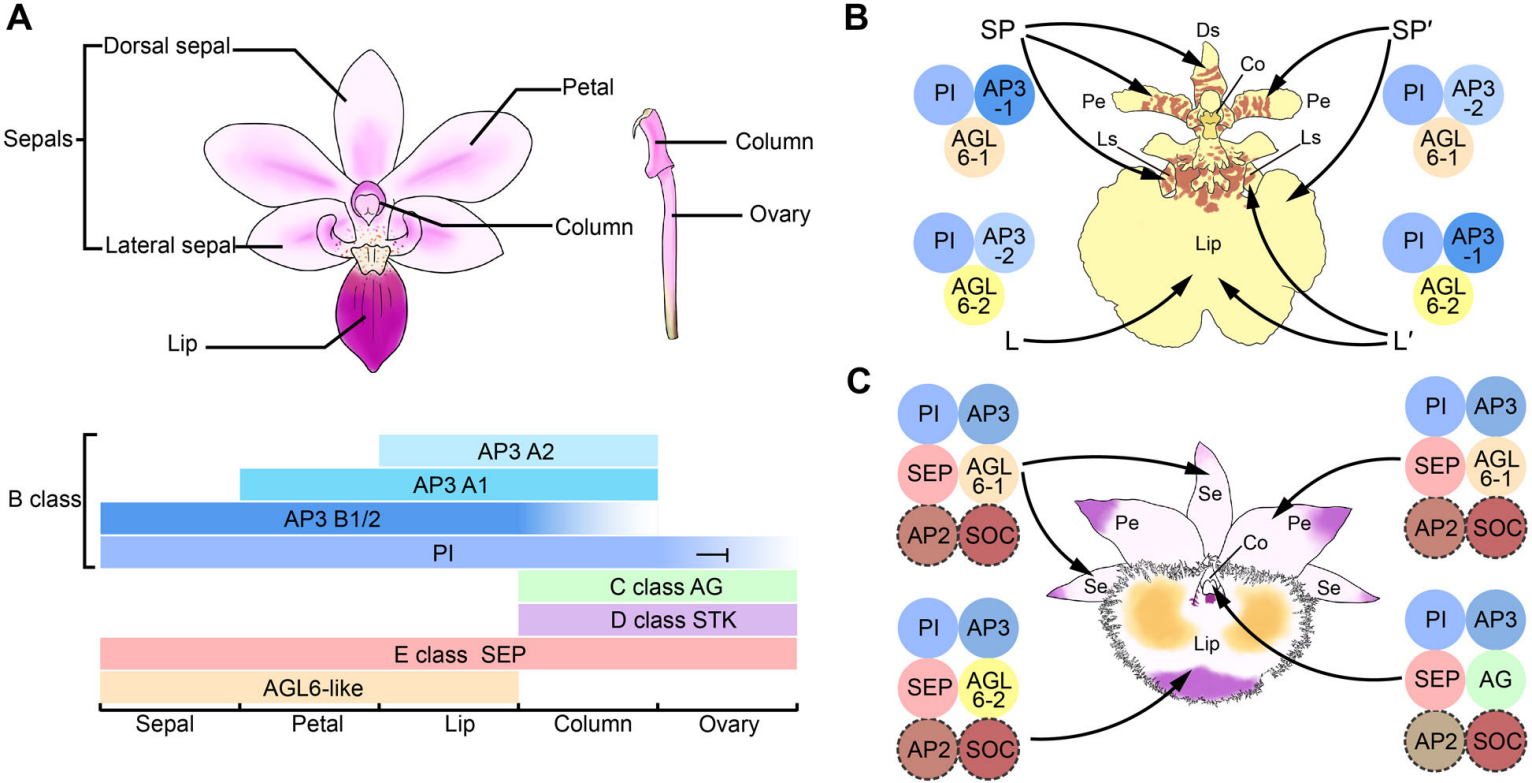
Integrated model of orchid floral organ identity, incorporating the Homeotic Orchid Tepal (HOT) model, the extended Perianth code (P‐code) model, and the MADS‐box module of *Dendrobium devonianum* **(A)** A MADS‐box framework for orchid floral organ identity, summarized from the HOT (Homeotic Orchid Tepal) model and subsequent studies, highlighting B‐class genes (*AP3/PI*) together with *AGL6*‐like, E class (*SEP*), C class (*AG*), and D class (*STK*) genes. Gradient shading indicates weak or variable expression and a reduced contribution to organ identity rather than a deterministic role. The ⊣ symbol denotes a putative inhibitory role of PI in the ovule of ovary development, as suggested by previous studies. **(B)** Extended P‐code model, showing the potential contributions of four PI–AP3–AGL6 complexes—SP (OAP3‐1/OAGL6‐1/OPI), L (OAP3‐2/OAGL6‐2/OPI), SP′ (OAP3‐2/OAGL6‐1/OPI), and L′ (OAP3‐1/OAGL6‐2/OPI)—to sepal, petal, and lip identity and to finer‐scale morphological differentiation. **(C)** *D. devonianum* MADS‐box module, showing organ‐enriched expression modules of the *AP3/PI*–*AGL6*–*SEP* module and other candidate regulators (e.g., *AP1/AP2* and *SOC*) across floral organs. Circles denote co‐expression or candidate regulatory modules and do not necessarily represent a single protein complex; dashed outlines indicate putative upstream or co‐expressed regulators. Co, column; Ds, dorsal sepal; Lip, lip; Ls, lateral sepal; Pe, petal; Se, sepal.

MADS‐box genes are classified into five classes (A–E) based on function and expression patterns. Beyond the classical ABCDE model, several orchid‐specific models have been proposed, including the Orchid Code (Mondragón‐Palomino and Theißen, 2008), the Homeotic Orchid Tepal (HOT) model (Pan et al., 2011), the Perianth code (P‐code) model (Hsu et al., 2015a), and the MADS‐box models of *Cym. ensifolium* (Ai et al., 2021) and *D. devonianum* (Wang et al., 2025b) (Figure 5). These models explain orchid‐specific traits such as lip specialization and gynostemium formation.

The HOT model emphasizes that functional diversification of orchid B‐class genes, *PISTILLATA* (*PI*) and *AP3*, is shaped not only by lineage‐specific duplications but also by developmental timing. During early floral primordium formation, *AP3* genes are broadly expressed, conferring a shared basal petaloid program across perianth whorls. At the floral bud stage, spatially partitioned combinations of B‐class paralogs with *AGL6*‐like genes specify organ identity: *PI*, *AP3B,* and *AGL6*‐like primarily contribute to sepal identity, *PI*, *AP3A1*, *AP3B,* and *AGL6*‐like to petal identity, whereas the lip is preferentially defined by *PI* together with *AP3A1/2* and *AGL6*‐like (Pan et al., 2011). Column formation typically involves *PI* and *AP3A1/2* acting in concert with C/D‐class genes, while ovary and ovule/ovary development is mainly governed by C/D‐class genes together with E‐class factors (Chen et al., 2012; Pan et al., 2014; Tsai et al., 2014). The SEPALLATA (SEP) proteins (E class) act as “molecular glue factors” in higher‐order MADS‐box complexes, interacting with diverse partners to assemble diverse complexes and thereby providing combinatorial control of floral organ identity and morphogenesis (Pan et al., 2014; Ai et al., 2021; Cheng et al., 2022). Hsu et al. (2015a) proposed the P‐code model, in which perianth identity is encoded by two competing complexes: The SP complex (OAP3‐1 + OAGL6‐1 + OPI) specifying sepals and typical petals, and the L complex (OAP3‐2 + OAGL6‐2 + OPI) specifying lip identity. The extended P‐code further suggests that an L′ complex (OAP3‐1 + OAGL6‐2 + OPI) may regulate the lip and the lower parts of the lateral sepals, whereas an SP′ complex (OAP3‐2 + OAGL6‐1 + OPI) may contribute to finer patterning of petals and the lip (Figure 5B); this regulatory network has also been linked to pigmentation patterns, flowering time, and abscission (Hsu et al., 2021). The MADS‐box module of *D. devonianum* additionally reports broad expression of putative A‐class genes (*AP1/AP2* and *SOC*) across multiple perianth whorls (Figure 5C); however, available evidence generally supports that orchid A‐class homologs primarily function upstream in floral transition and floral meristem identity, rather than serving as core determinants of organ identity (Tsai et al., 2014; Teo et al., 2019; Yang et al., 2021; Madrigal et al., 2024).

Mutant studies have been instrumental in elucidating MADS‐box gene function. In *Phalaenopsis*, *PhAGL6a*, *PhAGL6b*, and *PhMADS4* were highly expressed in the lip and lip‐like organs, promoting lip specialization (Huang et al., 2015). *PeNAC67*, *PeKAN2*, and *PeSCL23* acted synergistically with B‐class genes to regulate petal‐to‐lip transformation (Xu et al., 2024). A ternary complex involving *AP3*, *SEP3*, and *AGL6* has been implicated in regulating lateral sepal variation (Qi et al., 2025). In *Oncidium*, downregulation of *OMADS5* caused homeotic conversion of petals and sepals into lip‐like structures, indicating its role as a repressor of lip identity (Chang et al., 2010). In *Cym. ensifolium*, reduced expression of C‐class *AGAMOUS* (*AG*) genes leads to multitepal flowers, while their ectopic expression resulted in column‐like petals. Overexpression of *AP3* and *AGL6* enhanced lip‐like traits in other floral organs (Ai et al., 2021).

Epigenetic regulation provides an additional layer of gene regulation that modulates transcriptional activity without altering the underlying DNA sequence, thereby contributing to the spatial and temporal control of developmental programs (Köhler et al., 2012). In orchids, emerging evidence suggests that epigenetic mechanisms, including DNA methylation, histone modification, and small RNA‐mediated pathways, contribute to floral trait diversification and developmental plasticity. DNA methylation has been shown to influence floral pigmentation through the regulation of key metabolic genes. Differential methylation in the promoter region of the chalcone synthase (*CHS*) gene suppresses its expression and leads to reduced anthocyanin accumulation in *Oncidium* cultivars, establishing a direct link between promoter methylation and floral color variation (Liu et al., 2012). In addition to DNA methylation, histone modifications also modulate the expression dynamics of floral identity genes. In *Phalaenopsis*, increased histone acetylation (H3K9K14ac) at the translation start site of *PeMADS4*, together with promoter‐mediated regulation, correlates with its elevated expression in the lip, suggesting that histone acetylation contributes to the organ‐specific optimization of B‐class gene expression (Hsu et al., 2014). At the post‐transcriptional level, small RNA pathways further refine floral patterning and organ growth. Integrated transcriptomic analyses in *Cymbidium* species have identified several conserved miRNA–target modules, including miR156–SPL, miR167–ARF, miR319–TCP, and miR396–GRF, which participate in regulating meristem determinacy, organ proliferation, and floral pattern formation, with the miR396–GRF module playing a prominent role in controlling floral organ growth and proportion (Yang et al., 2017, 2022). Consistently, a transcriptome‐wide regulatory analysis in *D. officinale* revealed extensive organ‐specific accumulation of small RNAs and complex miRNA–target interaction networks involving transcription factors, hormone signaling components, and *ARGONAUTE 1* (AGO1)‐associated pathways, highlighting the importance of small RNA‐mediated post‐transcriptional regulation in orchid floral development (Meng et al., 2016). Collectively, these findings indicate that epigenetic regulation operates in concert with transcriptional networks, particularly the MADS‐box‐based floral identity system, to fine‐tune gene expression programs and ultimately contribute to the remarkable diversification of floral morphology in Orchidaceae.

Emerging omics technologies have further advanced research on floral development. Liu et al. (2022) applied 10x Visium spatiotemporal transcriptomics to construct a cellular atlas of floral development in *Ph*. “Big Chili”, identifying cell types including inflorescence meristems, floral primordia, and mature organs (e.g., lip and column). Genome‐wide association study (GWAS) studies have emerged as a powerful tool for identifying SNPs and genes responsible for quantitative trait loci (QTL) under natural variation (Wang et al., 2024). Yang et al. (2023b) conducted a GWAS in *Cym. sinense* and identified *RABBIT EARS (RBE)* as a regulator of floral morphology; *CsRBE* influences floral homeotic genes (*CsAP3*, *CsPI*) and boundary regulators (*CsCUC*, *CsTCP*). During floral development, multi‐omics analyses in *Cym. sinense* detected a GA and ABA antagonistic hub that regulates dormancy release and meristem outgrowth, in line with auxin‐mediated protocorm, inflorescence, and perianth morphogenesis (Novak et al., 2014; Ahmad et al., 2024). Using scRNA‐seq and mass spectrometry imaging, Wang et al. (2025b) dissected the molecular mechanisms underlying flower color (MYB/bHLH‐regulated yellow spots and purple tips) and fringed lip margins (involving *GL1‐1*, *MADS6*, *KAS12*, *ABCG36*, *MYB305*, *NAC068*, and *BZIP12*) in *D. devonianum*. Their work further supports that class A/B/E genes regulate perianth identity across whorls, while class C/D genes govern column‐specific reproductive traits.

Orchid floral diversity arises from MADS‐box gene expansion/contraction and their dynamic regulation within the ABCDE model framework, providing an evolutionary and mechanistic basis for how exceptional floral disparity can emerge from an otherwise conserved developmental architecture. Although orchid floral developmental models (e.g., HOT and the P code) have been extensively investigated, the molecular basis of column, pollinium, and ovule/ovary development remains comparatively underexplored. Another key open question is which upstream regulatory layers control the spatiotemporal assembly and target selection of MADS‐box protein complexes, thereby specifying organ identity and tissue‐specific morphogenesis in orchids. Despite the presumed role of specialized pollination in driving orchid diversification, the coevolutionary links between floral morphology, pollinator‐mediated selection, and shifts in pollination systems remain poorly understood. Addressing these questions with cross‐species comparative functional genomics and emerging omics technologies coupled with interaction/perturbation assays should clarify how regulatory‐network evolution underpins the unique floral morphology of Orchidaceae.

#### Regulation of floral color

Floral color is a key visual signal in Orchidaceae for attracting pollinators and is critical for ecological adaptation and ornamental value. The remarkable diversity of orchid colors is primarily determined by the biosynthesis of two major pigments: water‐soluble anthocyanins (conferring red to purple hues) and lipid‐soluble carotenoids (responsible for yellow to orange tones), in some cases, chlorophyll may influence greenish coloration. These pathways are tightly regulated by transcription factors, such as R2R3‐MYB, bHLH, WD40 (which form the MBW complex), as well as AGL6 and B‐class MADS‐box proteins. The anthocyanin biosynthesis pathway (ABP) involves structural genes, including *CHS*, flavanone 3‐hydroxylase (*F3H*), dihydroflavonol 4‐reductase (*DFR*), anthocyanidin synthase (*ANS*), and UDP‐glycosyltransferases (*UGTs*), coordinated by the MBW complex to produce red‐purple pigmentation (Ramsay and Glover, 2005). Meanwhile, the carotenoid biosynthesis pathway (CBP) involves structural genes, such as phytoene synthase (*PSY*), phytoene desaturase (*PDS*), β‐carotene hydroxylase (*BCH*), and zeaxanthin epoxidase (*ZEP*), which contribute to yellow‐orange coloration (Watkins and Pogson, 2020). Additionally, SP complex indirectly influences spatiotemporal color patterns by regulating pigment deposition during floral development (Hsu et al., 2021).

The genus *Dendrobium* exhibits exceptional color diversity, with particular interest in the short blooming period of yellow flowers and region‐specific pigmentation. Genomic analysis of *D. chrysotoxum* revealed that during flower senescence, increased carotenoid production and degradation of lutein into trace amounts of abscisic acid (ABA) promote ethylene biosynthesis, resulting in short‐lived yellow flowers (Zhang et al., 2021b). In the yellow lip of *D. thyrsiflorum*, genes including *CrtP‐like*, *CrtH‐like*, and *ZDS‐like* were identified as key regulators of carotenoid biosynthesis (Chen et al., 2024c). In *D. nobile*, the loss of purple floral coloration appears to be mainly attributable to the coordinated downregulation of key structural genes involved in flavonoid and anthocyanin biosynthesis, such as *DnCHS*, *DnF3′H*, *DnDFR*, and *DnGT1*, thereby reducing pigment accumulation in white‐flowered lines (Qiu et al., 2023). ScRNA‐seq and mass spectrometry imaging in *D. devonianum* demonstrated that yellow patches are associated with high expression of carotenoid genes (e.g., *PSY*, *PDS*, *BCH*) in epidermal and vascular cells, while purple regions correlate with upregulated anthocyanin genes (e.g., *O*‐methyltransferase (*OMT*), leucoanthocyanidin dioxygenase (*LDOX*), *UGT72L*). MYB and bHLH transcription factors form a developmentally specific regulatory network, enabling spatiotemporal patterning of flower color (Wang et al., 2025b). Furthermore, ethylene and ABA signaling fine‐tune dynamic color changes by modulating pigment gene expression.

Dynamic color changes in orchids reflect sophisticated spatiotemporal regulation. In *Phalaenopsis*, three R2R3‐MYB genes, *PeMYB2*, *PeMYB11*, and *PeMYB12*, govern overall red pigmentation, red spotting, and texture patterning, respectively, demonstrating region‐specific anthocyanin deposition (Hsu et al., 2015b). In *Cattleya* hybrid “KOVA”, *RcPAP1* activates anthocyanin synthesis before anthesis, resulting in purple‐red tepals; after flowering, *RcPAP2* downregulates anthocyanins, leading to pale pink tepals, while *RcPCP1* promotes the accumulation of α‐carotene and lutein, forming yellow lip regions (Li et al., 2020). In *Cym. goeringii*, high expression of *BCH*, lycopene *ε*‐cyclase (*LCYE*), lycopene *β*‐cyclase (*LCYB*), carotenoid isomerase (*CRTISO*), and *PDS* promotes pale yellow flowers with purple‐red spots, whereas *ANS* and UDP‐glucose: flavonoid 3‐*O*‐glucosyltransferase (*UFGT*) drive anthocyanin accumulation (Sun et al., 2021). *CfMYB1* activates ABP genes and likely underlies post‐pollination lip color changes (Ma et al., 2024). In *Cym. lowianum*, anthocyanins (especially cyanidin‐3‐*O*‐glucoside) dominate floral color, with carotenoids and chlorophyll playing modulating roles. A regulatory network involving flavonoid 3′‐hydroxylase (*F3*′*H*), *BCH*, protoporphyrinogen IX oxidase (*HEMG*), magnesium chelatase subunit I (*CHLI*), and transcription factors such as *MYB308‐1*, *MYB111*, *PIF4‐2*, and *MYB14‐1* was identified. Notably, *MYB14‐1* interacts with *PIF4‐2* to coordinate anthocyanin and chlorophyll synthesis, shaping color patterns in the lip and across floral organs (Dong et al., 2025). These findings illuminate the molecular basis of color diversity in orchids and provide targets for precision breeding.

#### Floral scent biosynthesis and pollinator specificity

Floral scent serves as a crucial chemical signal in Orchidaceae for pollinator attraction, characterized by a complex blend of volatile organic compounds primarily comprising terpenoids, phenylpropanoids/benzenoids, and fatty acid derivatives. The production of these compounds reflects a long history of co‐evolution between orchids and their pollinators.

Genomic and transcriptomic studies have revealed that the synthesis of diverse scent profiles is associated with the expansion and upregulation of key gene families, including terpene synthases (TPS), jasmonic acid methyltransferase (JMT), and phenylpropanoid pathway genes (e.g., phenylalanine ammonia‐lyase (*PAL*), cinnamate 4‐hydroxylase (*C4H*), cinnamyl alcohol dehydrogenase 6 (*CAD6*)), along with enhanced precursor supply through the methylerythritol phosphate (MEP) and mevalonic acid (MVA) pathways (Figure 4; Ai et al., 2021; Sun et al., 2021; Zhang et al., 2024; Tu et al., 2025). In *Ph. bellina*, a bHLH transcription factor, *PbbHLH4*, has been identified as a master regulator of monoterpene biosynthesis; its heterologous expression in scentless orchids increased monoterpenoid production by 950‐fold (Chuang et al., 2018). Similarly, in *Cym. tracyanum*, multiple transcription factors, including *CtAP2/ERF1*, *CtbZIP1*, *CtMYB2*, *CtMYB3*, and *CtAP2/ERM4*, activate *TPS* genes to modulate the emission of mono‐ and sesquiterpenes. Scent release is subject to multilayered regulation, including transcriptional control, photoperiod/circadian rhythms, and phytohormone signaling (e.g., JA, ethylene). Specific transcription factors (e.g., bHLHs, MYBs) and circadian clock‐associated elements fine‐tune the temporal expression of *TPS* and *JMT*, thereby governing diurnal scent emission patterns and intensity (Chuang et al., 2017; Jiang et al., 2025).

Beyond generalized fragrance, many orchids emit species‐specific volatiles as part of deceptive pollination strategies, effectively attracting particular pollinators. This high degree of specialization reinforces reproductive isolation and can promote rapid speciation even in the absence of geographical barriers. For instance, certain *Bulbophyllum* species produce methyl eugenol, raspberry ketone, and zingerone to attract fruit fly pollinators (Katte et al., 2020). A dedicated olfactory receptor, *BdorOR94b1*, enables male flies to detect methyl eugenol, which, upon ingestion, is converted into (E)‐coniferyl alcohol, a compound that enhances their attractiveness to females (Zhang et al., 2025). Another celebrated example is Darwin's orchid, *Angraecum sesquipedale*, known for its extremely long nectar spur (25–35 cm) and highly specialized pollinator relationship with the hawkmoth *Xanthopan praedicta*. Recent findings indicate that this orchid emits oximes (R_1_R_2_C═N─OH) as dominant scent compounds to attract crepuscular or nocturnal pollinators (Jiang et al., 2025). Notably, genomes of *Angraecum* and other orchids lack the cytochrome P450 family 79 (CYP79), which typically catalyzes oxime biosynthesis. Instead, a novel class of flavin monooxygenase (*FMO*) genes, termed *orchid oxime synthases 1–6*, appears to have evolved, indicating a lineage‐specific metabolic pathway.

The genus *Ophrys* exemplifies sexual deception: These orchids mimic female insects both visually (through red‐brown markings and epidermal textures on the lip) and chemically (via sex pheromone mimicry) to lure male pollinators. Their volatile bouquets are rich in long‐chain saturated and unsaturated hydrocarbons (Sedeek et al., 2013). Key biosynthetic enzymes include stearoyl‐ACP desaturases (*SAD1/2*), which contribute to alkene synthesis in *cis*‐regulatory contexts, while *SAD5/6* may operate in trans (Xu et al., 2012). Genomic analyses have further identified candidate genes involved in hydrocarbon and anthocyanin biosynthesis, along with transcription factors such as MYB, MADS, and TCP families (Sedeek et al., 2013). In *Ophrys sphegodes*, significant expansions were detected in gene families related to plant reproduction and floral development, particularly *SAD*, membrane‐bound fatty acid desaturases (*FADs*), and fatty acyl‐CoA reductases, which likely facilitated adaptive evolution and diversification through pollinator‐mediated selection (Russo et al., 2024).

Notably, floral color and scent may share biosynthetic precursors or regulatory pathways, suggesting coupled evolution in pollinator attraction strategies. The functional dissection of key biosynthetic genes has provided molecular evidence that subtle genetic variations can alter pollinator preference, thereby acting as ‘speciation genes’ that drive reproductive isolation. These findings offer robust molecular support for Darwin's classic hypothesis of coevolution.

## GENOMIC RESOURCES FOR ORCHID UTILIZATION AND CONSERVATION

### Medicinal active ingredient biosynthesis

Orchids have a long‐standing history of use as food and medicinal materials in human societies. Notable medicinal genera include *Dendrobium*, *Orchis*, *Anoectochilus*, *Bletilla*, *Bulbophyllum*, *Gastrodia*, and *Habenaria* (Teoh, 2019). The main chemical constituents of these taxa have been identified, including polysaccharides, phenolic compounds, alkaloids, terpenoids, and phenanthrenes (Ye et al., 2017; Wei et al., 2018; Ji et al., 2024b).

Gastrodin, a phenolic glycoside, is a principal functional constituent of *Ga. elata*. Pharmacological studies have indicated its therapeutic potential in neurological, cardiovascular, endocrine, and hepatic disorders (Guo et al., 2024). Integrated metabolomic and transcriptomic evidence supports a biosynthetic model in which gastrodin accumulation is mainly regulated by precursor supply and terminal glycosylation (Shan et al., 2021; Wang et al., 2025c). The abundances of gastrodin and its related intermediates, including *p*‐hydroxybenzyl alcohol (HBA) and *p*‐hydroxybenzaldehyde, are closely associated with carbon allocation through phenylalanine‐derived phenylpropanoid metabolism (Shan et al., 2021). In this context, coordinated expression of phenylpropanoid‐pathway genes and downstream alcohol dehydrogenase and glycosyltransferase genes is suggested to modulate HBA availability, thereby influencing the substrate pool for gastrodin biosynthesis; moreover, variation in gastrodin and HBA levels has been linked to concerted changes in glycosyltransferase gene families, highlighting candidate UDP‐glycosyltransferases potentially involved in HBA glycosylation (Wang et al., 2025c). Evidence from comparative transcriptomics suggests that developmental transitions of the tuber, as well as mycorrhizal interactions, are accompanied by marked transcriptional reprogramming of candidate monooxygenase or *CYP* genes and glycosyltransferase genes, consistent with a regulatory role of oxidation steps and terminal glycosylation in gastrodin biosynthesis (Tsai et al., 2016).

*Dendrobium* orchids are among the most widely utilized edible and medicinal orchids because they are rich in polysaccharides that have been reported to exhibit immunomodulatory, antioxidant, anti‐inflammatory, anti‐tumor, hypoglycemic, hepatoprotective, and gastrointestinal protective activities (Wei et al., 2018; Niu et al., 2021). The polysaccharides enriched in stems and leaves of *D. officinale* are mainly composed of mannose, glucose, and galactose. The biosynthesis of mannose‐containing polysaccharides (MCPs) is initiated by the diversion of carbon flux from primary sugar metabolism into the hexosamine biosynthetic route and the mannose‐metabolism branch: Fructose‐6‐phosphate is converted to mannose‐6‐phosphate by phosphomannose isomerase (PMI), subsequently to mannose‐1‐phosphate by phosphomannomutase (PMM), and then to GDP‐mannose by GDP‐mannose pyrophosphorylase (GMP), thereby establishing a pivotal precursor‐supply node for mannan production (Scheper et al., 2023).

Pathway annotation based on a chromosome‐level reference genome further identified 67 genes implicated in polysaccharide biosynthesis. Notably, fructose and mannose metabolism was significantly enriched, and salicylic acid (SA) induction was accompanied by upregulation of genes encoding key enzymes, such as hexokinase (HK) and mannose‐6‐phosphate isomerase (MANA), suggesting that this branch constitutes a set of candidate targets for regulating MCP accumulation (Niu et al., 2021). Polymerization of the mannan backbone is primarily mediated by cellulose synthase‐like A (CSLA) enzymes, and eight *DoCSLA* genes showed high expression in stems and are inducible under abiotic stresses, implying that environmental cues can modulate MCP yield through transcriptional regulation of polymerizing enzymes (He et al., 2015, 2017a). *DoGMP1* has been identified as a central regulatory determinant. Its transcription is positively activated by the WUSCHEL‐related homeobox transcription factor *DoWOX4*, but negatively modulated by the ethylene response factor *DoERF5*, collectively influencing mannan accumulation (He et al., 2017b; Zeng et al., 2025). GDP‐mannose is then transported into the Golgi lumen via the Golgi‐localized GDP‐mannose transporters *DoGMT1–3*, thereby providing activated sugar donors for polysaccharide assembly (Yu et al., 2018). Post‐polymerization modification is also integral to MCP properties: *O*‐acetylation mediated by the TRICHOME BIREFRINGENCE‐LIKE (TBL) family correlates with water‐soluble polysaccharide levels, with *DoTBL34/35* and related members showing positive associations with *O*‐acetyl group abundance and water‐soluble polysaccharide (WSP) accumulation during stem maturation (Si et al., 2022). In addition, sugar transport systems contribute to precursor partitioning, as several *DoSWEET* genes display tissue‐specific and stress‐responsive expression patterns, and gene silencing markedly reduces MCP levels (Li et al., 2025b). Collectively, MCP biosynthesis in *D. officinale* is governed by a multilayered regulatory hierarchy spanning precursor supply, activated‐donor transport, polymerization, post‐synthetic modification, and transport‐driven allocation.

Recent studies have begun to elucidate the biosynthetic mechanisms underlying other medicinally active ingredients. For bibenzyls and phenanthrenes, which are characteristic phenolic scaffolds in *Dendrobium*, transcriptome‐guided pathway reconstruction has prioritized type III polyketide synthases (bibenzyl synthases) and downstream oxidative tailoring enzymes as key determinants of product diversification (Adejobi et al., 2021; Zhou et al., 2024). Terpenoid pathways have likewise advanced from annotation to mechanism. *DoECS* has been functionally identified as an (E)‐*β*‐caryophyllene synthase, and MYB regulators have been implicated in its transcriptional control, linking gene regulation to terpene output (Lv et al., 2022). More directly, the *AP2/ERF* transcription factor *DoAP2/ERF89* binds the promoter of the terpene synthase gene *DoPAES* and activates *β*‐patchoulene biosynthesis, providing a clear transcription factor–target gene module for terpenoid regulation in *D. officinale* (Li et al., 2024b). The alkaloid biosynthesis of *D. officinale* was inferred to be primarily regulated through salicylic acid–responsive transcriptional activation of the MVA and MEP pathways and terpenoid‐backbone biosynthesis genes, thereby enhancing precursor supply for downstream sesquiterpene alkaloids and terpenoid indole alkaloids (Niu et al., 2021). In *Anoectochilus roxburghii*, mycorrhizal association promotes flavonoid accumulation and is accompanied by upregulation of CHS, indicating that symbiosis can act as a biologically relevant regulatory layer shaping medicinal quality (Zhang et al., 2020).

Collectively, research on the polysaccharide biosynthetic pathway in *D. officinale* has become relatively systematic, providing a useful reference framework for dissecting medicinal metabolite formation in Orchidaceae. Nevertheless, for many other medicinal orchids, mechanistic inference still relies heavily on correlative multi‐omics signals, whereas direct causal evidence remains limited. Key gaps include incomplete resolution of pathway topology for several compound classes, insufficient identification of rate‐limiting steps and metabolite‐transport processes, and a paucity of *in vivo* functional validation for key transcription factors and other regulatory components that likely underpin chemical diversification. Looking forward, deeper mechanistic elucidation of edible and medicinal constituents in orchids can inform germplasm selection, quality‐trait breeding, and the development of sustainable production systems, thereby reducing harvesting pressure on wild populations while safeguarding the evolutionary potential and chemical biodiversity of Orchidaceae.

### Genomics‐assisted conservation of Orchidaceae

Orchidaceae is among the most threatened plant families worldwide, owing to habitat fragmentation, over‐collection, illegal trade, and disruption of mycorrhizal and pollination networks (Hinsley et al., 2018). As of 2025, only 2,123 orchid species have been assessed for the International Union for the Conservation of Nature (IUCN) Red List, representing just 7.0% of the family (Fay et al., 2025). The entire family is regulated under the Convention on International Trade in Endangered Species (CITES) (Hinsley et al., 2018). Together, these patterns highlight both the scale of extinction risk across orchids and the urgency of developing more effective conservation frameworks. Current research in orchid conservation biology has therefore focused on four major themes: Threat assessment and risk identification, *in situ* and *ex situ* conservation, mycorrhizal symbiosis and reintroduction, and the use of genetic and genomic data to delimit conservation units (Fay et al., 2025; Hartvig et al., 2025).

Field‐based surveys of germplasm resources and conservation assessment remain the foundation of orchid conservation. At present, orchid assessments are relatively advanced in Europe and parts of Africa, whereas major diversity hotspots, particularly tropical Asia and South America, remain substantially under‐evaluated (Wraith et al., 2020; Vitt et al., 2023). In this context, Zhan et al. (2024) argued that orchid conservation assessment should move beyond conventional species occurrence surveys toward a multidimensional framework integrating taxonomic, phylogenetic, and functional diversity. Such an approach enables more precise identification of conservation priorities and irreplaceable lineages. Current orchid conservation practice has increasingly converged on four core strategies: *in situ* protection, *ex situ* conservation, reintroduction and population restoration, and long‐term seed and germplasm banking.

Among these approaches, mycorrhizal symbiosis has emerged as a defining theme in orchid conservation biology. Unlike most angiosperms, orchid seeds are minute and lack endosperm, and their germination and early establishment depend on compatible mycorrhizal fungi. As a result, the success of both ex situ propagation and field reintroduction is closely tied to plant–fungus compatibility. Recent integration of population genomics with root‐associated and endophytic fungal community profiling has provided a more complete view of orchid–microbe interactions and their conservation relevance (Hartvig et al., 2025). Building on this framework, researchers have increasingly screened fungal isolates obtained from protocorms or germinating seeds as candidate symbionts for reintroduction programs (Zhao et al., 2021, 2024b). Experimental evidence further shows that inoculation with germination‐promoting fungi can substantially increase seed germination and seedling establishment of terrestrial orchids under natural conditions, leading to the development of the concept of fungus‐assisted reintroduction (Shao et al., 2022). At broader scales, global analyses indicate that orchid mycorrhizal communities are shaped primarily by trophic mode, geography, and environmental niche, rather than by phylogenetic relatedness alone (Wang et al., 2025d). This has important implications for conservation: Protecting orchid populations in isolation is unlikely to be sufficient if the associated fungal resource pools and the ecological filters that structure them are not also maintained. Orchid reintroduction has therefore shifted from simple transplantation of propagated individuals toward integrative strategies that combine symbiotic propagation, selection of effective mycorrhizal partners, and restoration of suitable habitat conditions.

China has developed a comparatively comprehensive research framework for orchid conservation. Current estimates indicate that the country contains 1,708 species in 181 genera; of these, ca. 1,100 species are covered by national nature reserves and ca. 800 have been incorporated into living collections in major botanical gardens. In addition, field pollination biology has been investigated in 74 species, and 29 species have been subjected to population‐level analyses of genetic diversity or spatial genetic structure (Zhou et al., 2021). Overall, future work should prioritize field surveys and threat assessment in tropical diversity hotspots, deepen mechanistic understanding of orchid–mycorrhizal interactions and their environmental sensitivity, and more effectively link conservation assessment with symbiotic propagation and reintroduction practice. Such integration will be essential for improving the long‐term persistence of threatened orchid species.

## PROSPECTS

In recent years, orchid research has seen substantial advancements, particularly over the past decade, with the widespread adoption of high‐throughput sequencing technologies enabling the assembly of numerous genomes and phylogenomic datasets. These efforts have clarified subfamily relationships, elucidated ancient origins tied to major geological events, and uncovered molecular bases for traits like epiphytism, mycoheterotrophy, and deceptive pollination through gene family analyses and multi‐omics integrations. Studies have increasingly focused on integrating transcriptomics and metabolomics to dissect symbiotic interactions and floral development, while biogeographic models have highlighted diversification hotspots in tropical regions.

Looking ahead, the field promises further breakthroughs via cutting‐edge technologies and interdisciplinary collaborations. The T2T genome assembly, paired with long‐read sequencing platforms, will produce complete, haplotype‐phased references to tackle complexities like heterozygosity and repetitive regions. Multi‐omics fusion, encompassing genomics, transcriptomics, metabolomics, proteomics, and epigenomics, will yield comprehensive views of regulatory networks governing floral deception, mycorrhizal dependencies, and photosynthetic adaptations, supported by single‐cell and spatial transcriptomics for fine‐scale cellular insights. Open resource sharing through databases will promote global phylogenomics and pan‐genome initiatives across orchid lineages. However, challenges remain, including reconciling cytonuclear phylogenetic conflicts in heterotrophic groups, assessing transposable element impacts on rapid speciation, and predicting diversification under climate change scenarios. Addressing these will deepen evolutionary knowledge, bolster conservation in vulnerable hotspots, and advance applications in horticulture and pharmacology, fostering a synergy between basic science and practical benefits.

## CONFLICTS OF INTEREST

The authors declare no conflicts of interest.

## AUTHOR CONTRIBUTIONS

Z.J.L., F.X.Y., and M.Y.Z. reviewed the manuscript. M.Y.Z., C.Y.Z., L.W., and J.G. drafted the manuscript. M.Y.Z., C.Y.Z., and L.W. drew the figures. W.Y., D.H.P., S.L., F.X.Y., and Z.‐J.L. revised the manuscript. All authors discussed and approved the manuscript.
